# Supplementary material for: Global systematic review with meta-analysis shows that warming effects on terrestrial plant biomass allocation are influenced by precipitation and mycorrhizal association
Source: Nat Commun. 2022 Aug 20;13:4914. doi: 10.1038/s41467-022-32671-9 (PMC9392739; doi:10.1038/s41467-022-32671-9)
Supplement: Supplementary file 1 — Supplementary Information [file 41467_2022_32671_MOESM1_ESM.pdf]

Supplementary Information

I) Supplementary Figures

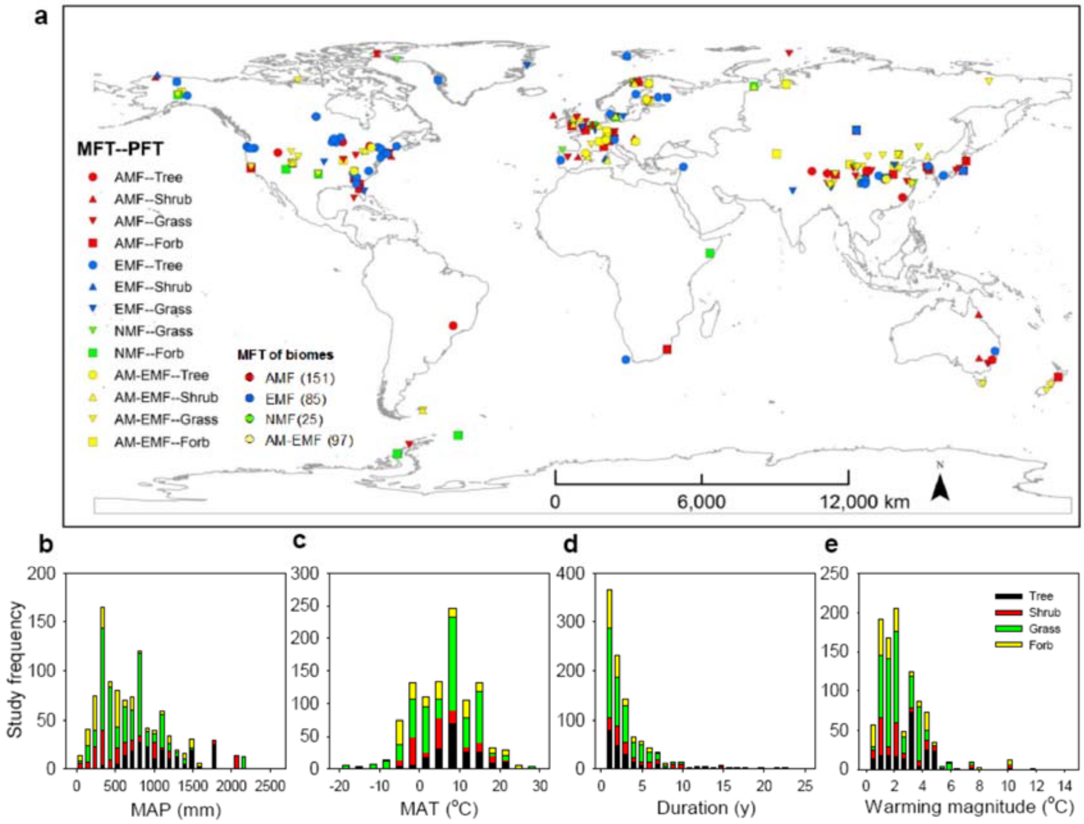

Supplementary Figure 1 **Site location and frequency of warming studies included in this meta-analysis.** (**a)** Site location of warming studies included in this study. (**b-d)** The frequency of mean annual precipitation (MAP, mm, **b**), temperature (MAT, °C, **c**), warming duration (year, **d**) and magnitude (°C, **e**) in the selected studies. MFT--PFT indicates a biome with dominant root symbiosis of mycorrhizal fungi type (MFT, i.e., AMF: arbuscular mycorrhizal fungi; EMF: ectomycorrhizal fungi; NMF: non-mycorrhizal fungi; AM-EMF: mixed arbuscular and ecotomycorrhizal fungi), and dominant plant functional type (PFT, i.e., tree, shrub, grass, forb). The numbers in the parentheses were the study frequency in each MFT in panel **a**.

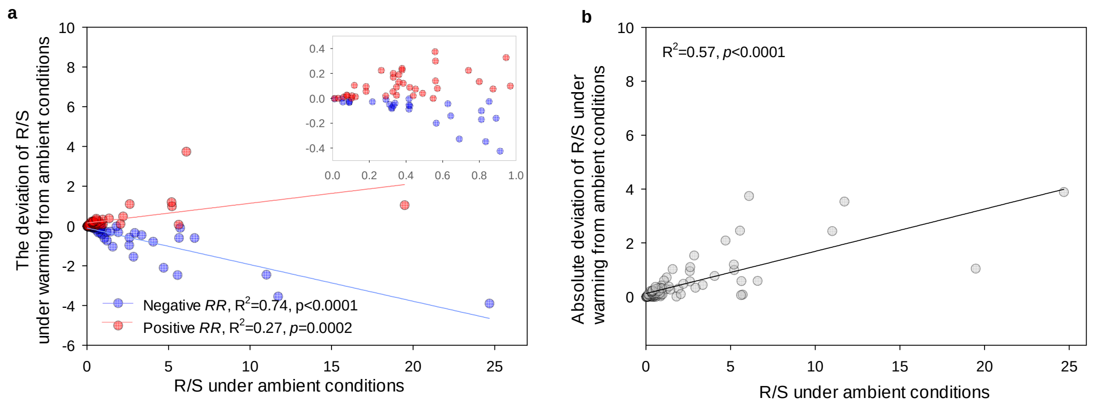

Supplementary Figure 2 The deviation (a) and absolute deviation (b) of root: shoot ratio (R/S) under warming from ambient conditions along with R/S under ambient conditions. The insert panel in **a** shows the data points with R/S lower than 1 under ambient conditions. Negative and Positive *RR* indicate the points with negative and positive response ratios of R/S, respectively. The *p*-values were calculated from two-tailed tests.

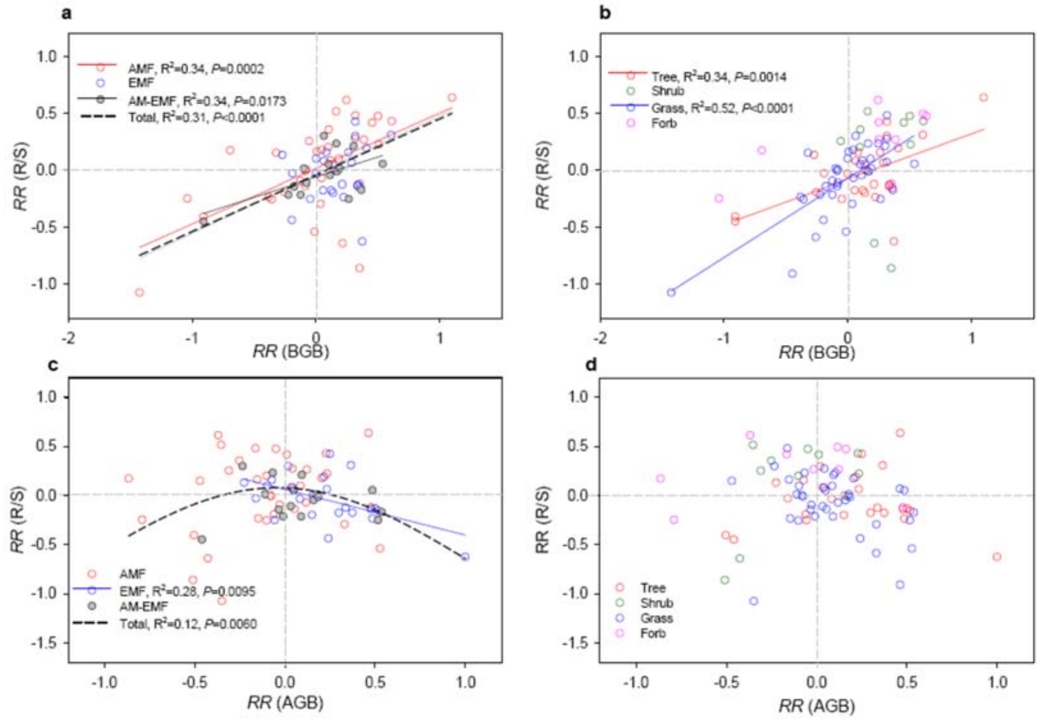

Supplementary Figure 3 The relationship between response of below- [*RR*(BGB), a and b] and aboveground biomass [*RR*(AGB), c and d] with that of root: shoot ratio (R/S). AMF, EMF, AM-EMF, and total indicated biomes with dominant root symbiosis of arbuscular mycorrhizal (AMF), ectomycorrhizal fungi (EMF), and mixed AMF and EMF (AM-EMF), and across all studies (Total), respectively. The *p*-values in panels **a**, **b** and **c**, were calculated from two-tailed tests.

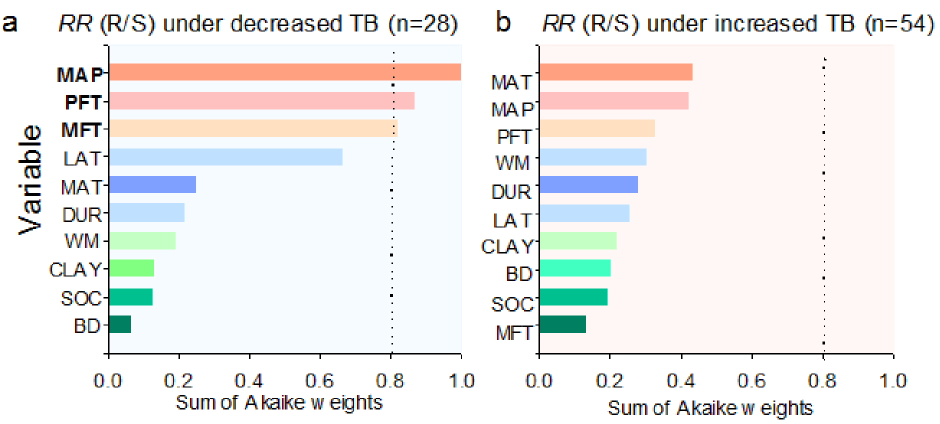

Supplementary Figure 4 Model-averaged importance of the predictors for response of root: shoot ratio [*RR*(R/S)] to warming based on studies with decreased and increased plant total biomass (TB, a and b). The variables with an importance value >0 were considered as essential predictors. The important value is based on the sum of Akaike weights derived from the model selection using corrected Akaike's information criteria. PFT: plant functional type; MFT: mycorrhizal fungi type; MAP: mean annual precipitation; MAT: mean annual temperature; LAT: latitude; CLAY: the proportion of clay in soil; BD: bulk density; SOC: soil organic carbon; WM: warming magnitude; DUR: warming duration.

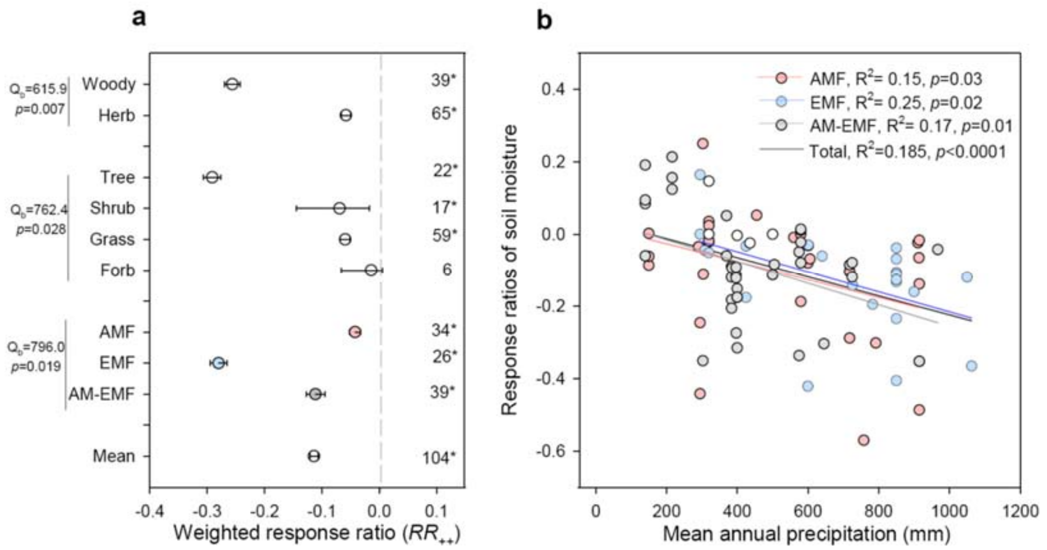

Supplementary Figure 5 Weighted response ratio (*RR*++) of soil moisture for different biomes (a), and the correlation between mean annual precipitation (mm) and response of soil moisture (b). Tree, Shrub, Grass, and Forb are biomes dominated by trees, shrubs, grasses, and forbs, respectively; AMF, EMF and AM-EMF were biomes with dominant root symbiosis of arbuscular mycorrhizal (AMF), ectomycorrhizal fungi (EMF), mixed AMF and EMF (AM-EMF), respectively. In panel **a**, the centre of circles and error bars indicate the mean and 95% confidence intervals (CI) of the weighted response ratio. If the 95% CI did not overlap with zero, it was then considered as an indication of a significant difference. Red and blue circles were used to indicate a significant warming-induced positive and negative response, respectively. The numbers in panels were the sample sizes, and the symbol * indicated a significant increase and decrease of variables under warming conditions in comparison with control (*p*-value<0.05). If the 95% CI of two effect sizes didn't overlap, it was then considered as an indication of a significant difference. All the *p*-values in panel **b** were calculated from two-tailed tests.

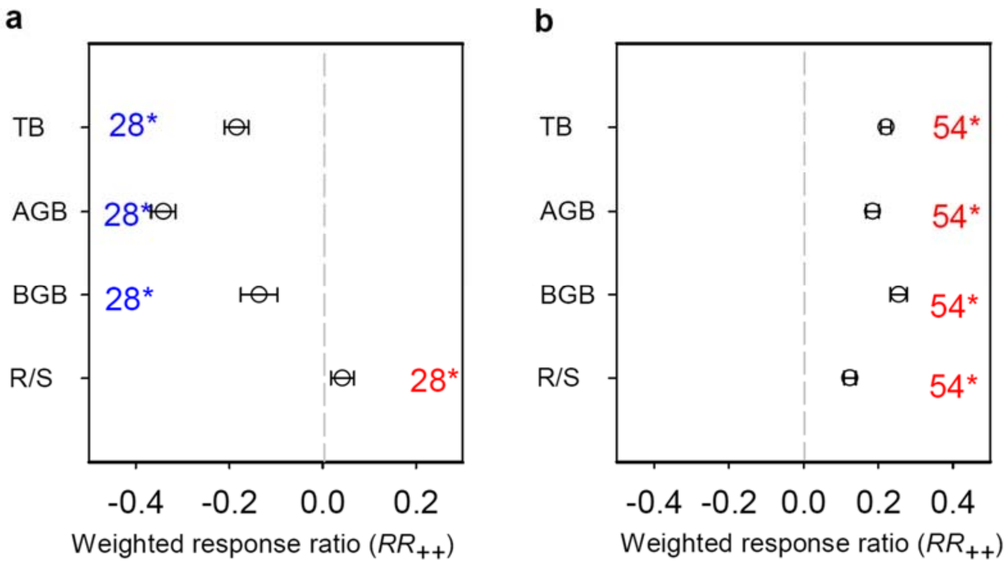

Supplementary Figure 6 Weighted response ratio (*RR*++) of plant total biomass (TB), above- and belowground biomass (AGB and BGB), and root: shoot ratio (R/S) in sites with a warming-induced decrease (a) or increase (b) of TB. The centre of circles and error bars indicated the mean and 95% confidence intervals (CI) of the weighted response ratio. If the 95% CI did not overlap with zero, a significant warming effect was considered. The numbers in panels were the sample sizes, and * with blue and red colour indicated a significant increase and decrease of variables under warming conditions in comparison to ambient conditions (p-value<0.05). If the 95% CI of two effect sizes didn't overlap, it was then considered as an indication of a significant difference.

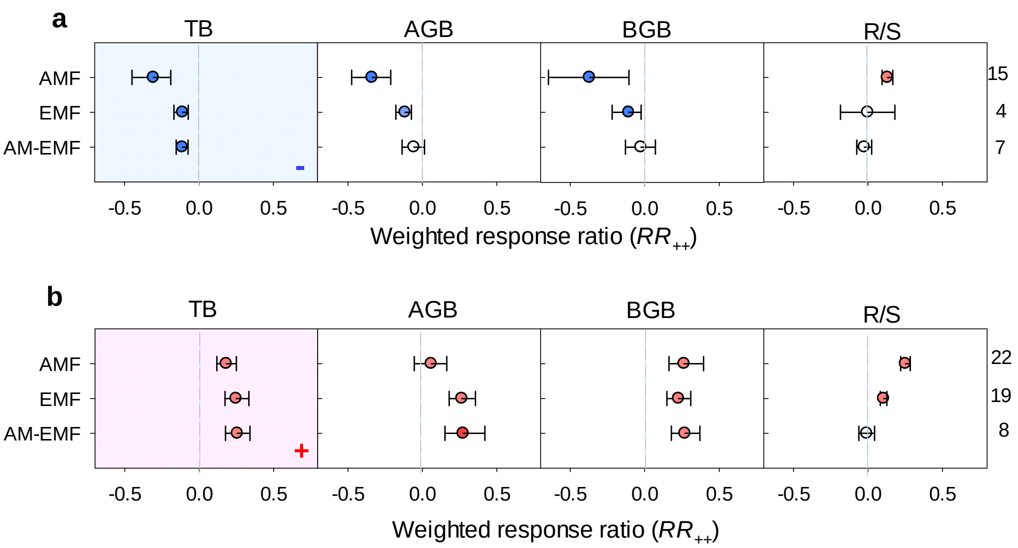

Supplementary Figure 7 Weighted response ratio of plant total biomass (TB), above- and belowground biomass (AGB and BGB), and root: shoot ratio (R/S) for different biomes in sites with a warming-induced decrease (a) or increase (b) of TB. AMF, EMF, and AM-EMF indicate the biomes dominated with root symbioses of arbuscular mycorrhizal (AMF), ectomycorrhizal fungi (EMF), mixed AMF and EMF (AM-EMF), respectively. The numbers on the right of the panels **a** and **b** were the sample sizes. The centre of the circle indicated the mean of the weighted response ratio, and the error bars indicated 95% confidence intervals (CI). If it did not overlap with zero, a significant warming-induced response was considered. Based on the two-tailed tests, the blue and red circles indicate increase and decrease under warmed conditions in comparison to ambient conditions for a given response variable (p-value<0.05).

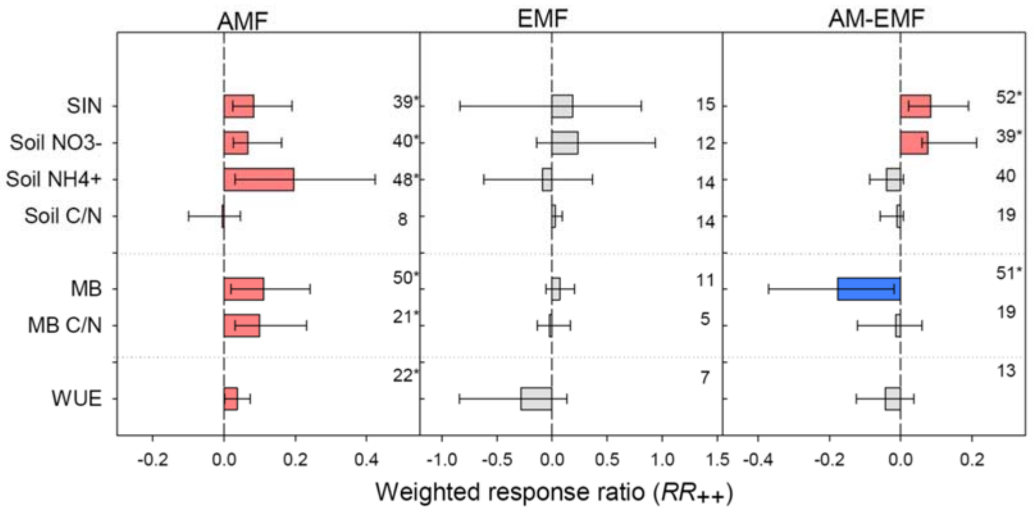

Supplementary Figure 8 Weighted response ratio of soil inorganic nitrogen (SIN), soil NO_3^-, soil NH_4^+, soil C/N, microbial biomass (MB), microbial C/N (MB C/N), and water use efficiency (WUE) for different biomes. AMF, EMF, and AM-EMF indicated the biomes dominated by root symbioses of arbuscular mycorrhizal (AMF), ectomycorrhizal fungi (EMF), mixed AMF and EMF (AM-EMF), respectively. The numbers in panels were the sample sizes; the column and bars indicate mean and 95% confidence intervals (CI), respectively. The symbol * indicate a significant effect of warming (*p*-value<0.05).

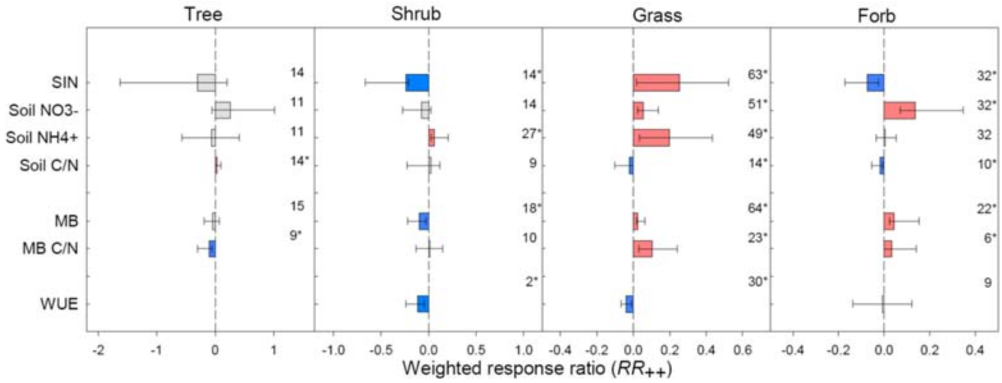

Supplementary Figure 9 Weighted response ratio (*RR*++) of soil inorganic nitrogen (SIN), soil NO_3^-, soil NH_4^+, soil C/N, microbial biomass (MB), microbial C/N (MB C/N), and water use efficiency (WUE) for biomes dominated by grasses, forbs, trees, or shrubs. The numbers in panels were the sample sizes. The column and bars indicate the mean and 95% confidence intervals (CI), respectively. The symbol * indicates a significant effect of warming (*p*-value<0.05).

II) Supplementary Tables

Supplementary Table 1 Ranges of predictor variables for warming responses of biomass allocation.

Predictor variables	Unit	Range	Median
Mean annual precipitation (MAP)	mm	27 ~ 2400	636.2
Mean annual temperature (MAT)	ºC	-20 ~30	7.4
Warming magnitude (WM)	ºC	0.26 ~12	2.0
Experimental duration (DUR)	GS or year	One GS ~ 25	2.0
Latitude	º	-68.02~78.9	41.3
Soil organic carbon (SOC)	g/kg	0.17 ~ 33.63	1.2
Bulk density (BD)	g/cm^3	0.76 ~ 1.66	1.4
Clay	%	4 ~ 62	19.0

MAP: with spatial resolution of 0.5º; MAT: with spatial resolution of 0.5º; GS: growing season; SOC: 0-30 cm depth from version 1.2 HWSD, with spatial resolution of 6%-46% 30 arc-second; BD: 0-30 cm depth from IGBP-DIS, with spatial solution of 0.08333º; Clay: soil clay content, 0-30 cm depth, from version 1.2 HWSD, with spatial resolution of 6%-46% 30 arc-second.

Supplementary Table 2 Blomberg's K values for response of root: shoot ratio [*RR*(R/S)], plant total biomass [*RR*(TB)], above- and belowground biomass [*RR*(AGB) and *RR*(BGB)] of 164 vascular plant species to warming.

Variables	*Blomberg's K*	***p*-values**
RR(R/S)	1.713	**0.001**
RR(TB)	0.104	**0.024**
RR(AGB)	0.065	0.479
RR(BGB)	0.031	0.848

Bold values (*p*-value<0.05, or <0.01) denote that both *RR*(R/S) and *RR*(TB) under warmed conditions exhibit a significant phylogenetic signal.

Supplementary Table 3 Stepwise linear regression for response of root: shoot ratio [*RR*(R/S)] with predictor variables for biomes with different plants.

Groups	Equation	R^2	*p*-values
AMF	*RR*(*R/S*) = -0.001MAP + 0.591	0.353	<0.001
	RR(*R/S*) = -0.001MAP - 0.033MAT + 0.803	0.481	<0.001
EMF	*RR*(*R/S*) = -0.032MAT + 0.284	0.328	0.003
	RR(*R/S*)= -0.029MAT - 0.067WM + 0.419	0.450	0.002
Woody plant	*RR*(*R/S*) = -0.001MAP +0.517	0.304	<0.001
	RR(*R/S*) = -0.001MAP -0.044DUR+0.667	0.408	<0.001
Herb	*RR*(*R/S*) = 0.030DUR -0.174	0.171	0.003
	RR(*R/S*) = 0.028DUR+0.000MAP+0.110	0.312	<0.001
	RR(*R/S*) = 0.029DUR-0.001MAP+0.016MAT +0.187	0.386	<0.001
Tree	*RR*(*R/S*) = -0.001MAP +0.508	0.271	0.003
	RR(*R/S*) = -0.001MAP +0.008ClAY+0.666	0.382	0.002
Shrub	*RR*(*R/S*) = -0.059MAT + 0.539	0.632	0.006
	RR(*R/S*) = -0.077MAT -4.884BD+ 7.286	0.966	<0.001
Forb	*RR*(*R/S*) = -0.001MAP +0.178	0.805	<0.001

AMF: biomes dominated by root symbiosis of arbuscular mycorrhizal; EMF: biomes dominated by root symbiosis of ectomycorrhizal fungi; Clay: soil clay content (%); BD: bulk density (g/cm^3); SOC: soil organic carbon (g/kg); LAT: latitude (°); MAT: mean annual temperature (°C); MAP: mean annual precipitation (mm); DUR: experimental duration (year); WM: warming magnitude (°C). The *p*-values were calculated from two-tailed tests.

Supplementary Table 4 Response heterogeneity (Q_b) of root: shoot ratio (R/S), plant total biomass (TB), above- and belowground biomass (AGB and BGB) between different groups.

**Variables**	**Group 1**	**Group 2**	Q_b	***p*-values**
RR(R/S)	Field	Laboratory	40.152	0.422
RR(R/S)	Evergreen	Deciduous	17.393	0.494
RR(R/S)	Broad	Coniferous	44.032	0.146
RR(R/S)	Annual	Perennial	273.064	0.075
RR(R/S)	Ang.	Gym.	57.926	0.398
RR(R/S)	Monoculture	Mixed	35.914	0.538
RR(TB)	Field	Laboratory	38.211	0.555
RR(TB)	Evergreen	Deciduous	25.901	0.407
RR(TB)	Broad	Coniferous	25.827	0.457
RR(TB)	Annual	Perennial	45.078	0.268
RR(TB)	Ang.	Gym.	21.865	0.440
RR(TB)	Monoculture	Mixed	9.014	0.634
RR(AGB)	Field	Laboratory	11.526	0.457
RR(AGB)	Evergreen	Deciduous	193.949	0.058
RR(AGB)	Broad	Coniferous	55.779	0.470
RR(AGB)	Annual	Perennial	343.031	0.224
RR(AGB)	Ang.	Gym.	31.901	0.633
RR(AGB)	Monoculture	Mixed	425.553	0.147
RR(BGB)	Field	Laboratory	25.457	0.573
RR(BGB)	Evergreen	Deciduous	0.724	0.894
RR(BGB)	Broad	Coniferous	44.524	0.333
RR(BGB)	Annual	Perennial	109.976	0.112
RR(BGB)	Ang.	Gym.	138.519	0.075
RR(BGB)	Monoculture	Mixed	21.417	0.486

RR: response ratio; Field and Laboratory: groups with studies conducted in filed and laboratory, respectively; Ang. and Gym.: groups with dominant angiosperms and gymnosperms in biomes, respectively; Monoculture and Mixed: groups with monoculture and mixed community, respectively. The *p*-values were calculated from two-tailed tests.

Supplementary Table 5 The results of ANOVA showing the effects of biomes, plant functional types (PFTs), and mycorrhizal fungi types (MFTs) on responses of plant total biomass (TB), above- and belowground biomass (AGB and BGB), and root: shoot ratio (R/S) to warming.

**Sources**	**Variables**	**df**	**MS**	**F**	***p*-values**
Biomes	*RR*(TB)	4	0.271	1.088	0.366
PFTs	*RR*(TB)	3	0.526	2.113	0.102
MFTs	*RR*(TB)	2	0.520	2.089	0.129
Biomes×PFTs	*RR*(TB)	2	0.436	1.754	0.178
Biomes×MFTs	*RR*(TB)	4	0.332	1.334	0.262
PFTs×MFTs	*RR*(TB)	0	--	--	--
Biomes×PFTs×MFTs	*RR*(TB)	0	--	--	--
Biomes	*RR*(AGB)	4	0.200	1.863	0.118
PFTs	*RR*(AGB)	3	0.152	1.119	0.244
MFTs	*RR*(AGB)	2	0.582	5.421	**0.001****
Biomes×PFTs	*RR*(AGB)	4	0.112	1.044	0.385
Biomes×MFTs	*RR*(AGB)	2	0.029	0.271	0.763
PFTs×MFTs	*RR*(AGB)	1	0.095	0.888	0.347
Biomes×PFTs×MFTs	*RR*(AGB)	0	--	--	--
Biomes	*RR*(BGB)	5	0.547	5.282	**0.000*****
PFTs	*RR*(BGB)	3	0.645	6.226	**0.001****
MFTs	*RR*(BGB)	2	0.216	2.090	0.129
Biomes×PFTs	*RR*(BGB)	3	0.075	0.724	0.540
Biomes×MFTs	*RR*(BGB)	1	0.301	2.905	0.091
PFTs×MFTs	*RR*(BGB)	1	0.000	0.001	0.978
Biomes×PFTs×MFTs	*RR*(BGB)	0	--	--	--
Biomes	*RR*(R/S)	4	0.065	0.743	0.566
PFTs	*RR*(R/S)	3	0.187	2.131	0.104
MFTs	*RR*(R/S)	2	0.124	1.417	0.249
Biomes×PFTs	*RR*(R/S)	2	0.145	1.647	0.200
Biomes×MFTs	*RR*(R/S)	1	0.370	4.215	**0.044***
PFTs×MFTs	*RR*(R/S)	0	--	--	--
Biomes×PFTs×MFTs	*RR*(R/S)	0	--	--	--

RR: response ratio; Biomes including cropland, desert, forest, grassland, tundra, and wetland; Plant functional types including grass, forb, shrub, and tree; Mycorrhizal fungi types including AM, EM, and AM-EMF, which indicated the biomes with dominant root symbiosis of arbuscular mycorrhizal (AMF), ectomycorrhizal fungi (EMF), mixed AMF and EMF (AM-EMF), respectively. Symbols *, **, *** indicate statistical significance at p-value<0.05, <0.01, and <0.001, respectively. The p-values were calculated from one-tailed tests.

Supplementary Table 6 The results of ANOVA showing the effects of warming magnitude and warming duration on the response of root: shoot ratio (R/S) to warming.

Sources	Variables	df	MS	F	*p*-values
Warming magnitude (WM)	*RR*(R/S)	5	0.050	0.503	0.772
Warming duration (DUR)	*RR*(R/S)	5	0.059	0.597	0.702
WM × DUR	*RR*(R/S)	5	0.064	0.648	0.664
Warming magnitude (WM)	*RR*(TB)	5	0.189	0.708	0.618
Warming duration (DUR)	*RR*(TB)	5	0.244	0.912	0.476
WM × DUR	*RR*(TB)	17	0.150	0.560	0.915
Warming magnitude (WM)	*RR*(AGB)	5	0.132	0.873	0.500
Warming duration (DUR)	*RR*(AGB)	5	0.043	0.284	0.922
WM × DUR	*RR*(AGB)	19	0.103	0.681	0.836
Warming magnitude (WM)	*RR*(BGB)	5	0.052	0.356	0.877
Warming duration (DUR)	*RR*(BGB)	5	0.073	0.499	0.776
WM × DUR	*RR*(BGB)	18	0.227	1.554	0.087

RR: response ratio; The levels of subgroups of warming magnitude (WM, increased <1, 1-2, 2-3, 3-4, 4-5, >5 ^{o}C, respectively), and experimental duration (DUR, for <1, 1-2, 2-3, 3-4, 4-5, >5 years, respectively) of selected studies in the meta-analysis. The *p*-values were calculated from one-tailed tests.

Supplementary Table 7 The results of ANOVA showing the effects of warming methods (including open top chamber, infrared heater, soil heating cable, and greenhouse) and root/belowground biomass measurement (RM, including direct harvest, soil core, and ingrowth mesh bags) on responses of plant total biomass (TB), above- and belowground biomass (AGB and BGB), and root: shoot ratio (R/S) to warming.

**Sources**	**Variables**	**df**	**MS**	**F**	***p*-values**
Warming methods	*RR*(TB)	3	0.543	2.149	0.098
RM	*RR*(TB)	2	0.127	0.503	0.606
Warming methods ×RM	*RR*(TB)	5	0.284	1.123	0.352
Warming methods	*RR*(AGB)	3	0.091	0.666	0.574
RM	*RR*(AGB)	-	-	-	-
Warming methods ×RM	*RR*(AGB)	-	-	-	-
Warming methods	*RR*(BGB)	3	0.04	0.346	0.792
RM	*RR*(BGB)	2	0.078	0.675	0.514
Warming methods ×RM	*RR*(BGB)	2	0.076	0.662	0.52
Warming methods	*RR*(R/S)	3	0.168	1.426	0.242
RM	*RR*(R/S)	2	0.041	0.349	0.707
Warming methods ×RM	*RR*(R/S)	5	0.206	1.75	0.134

The *p*-values were calculated from one-tailed tests.

III) Supplementary Notes

PRISMA flow diagram. The exclusion of studies and underlying reasons are provided for each stage based on Page et al. (2020).

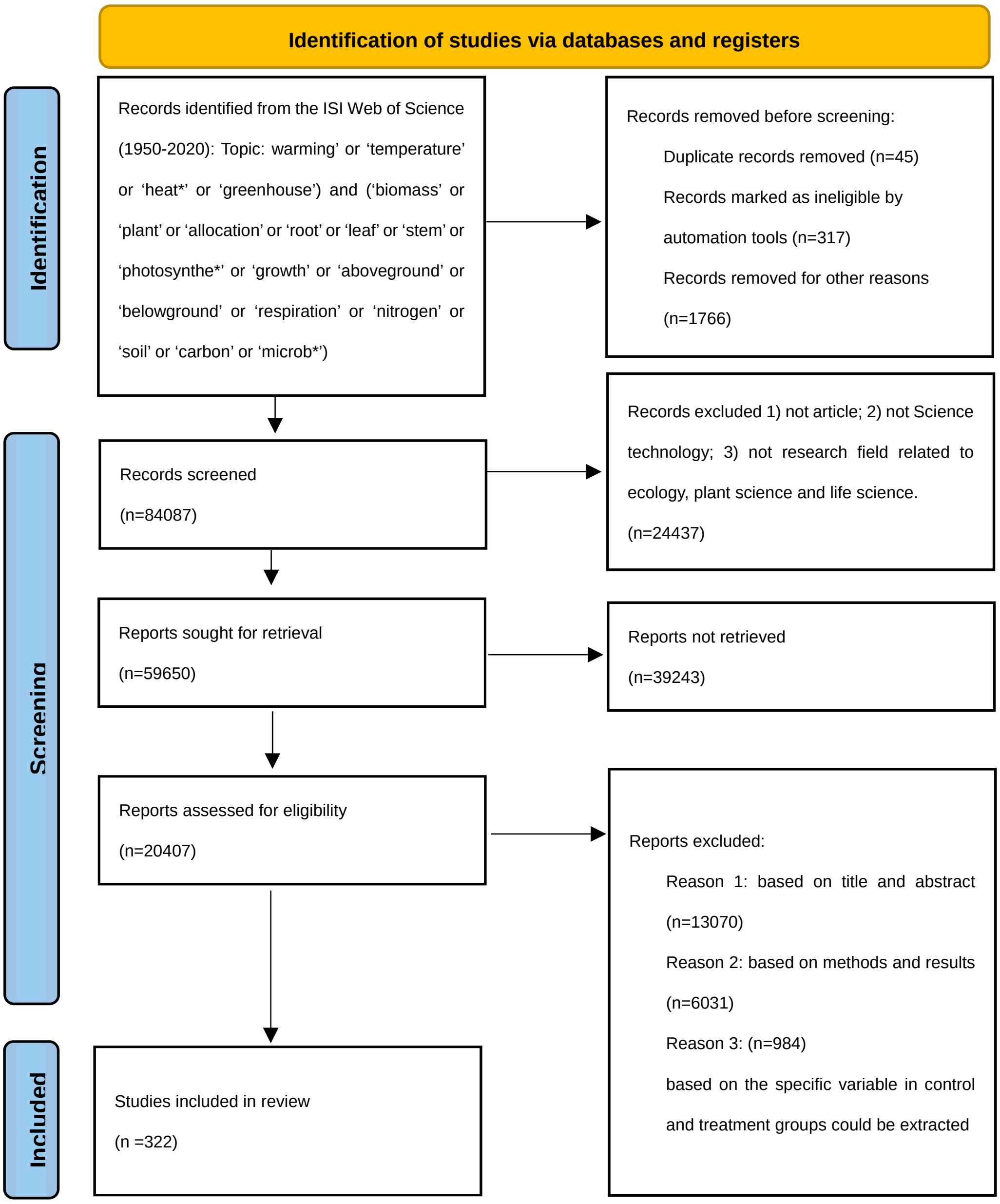

Page MJ, McKenzie JE, Bossuyt PM, Boutron I, Hoffmann TC, Mulrow CD, et al. The PRISMA 2020 statement: an updated guideline for reporting systematic reviews. BMJ 2021;372:n71. doi: 10.1136/bmj.n71

IV) Supplementary References

The list of 322 papers from which the data were extracted for this meta-analysis.

1 Abebe, A. *et al.* Growth, yield and quality of maize with elevated atmospheric carbon dioxide and temperature in north–west India. *Agriculture, Ecosystems & Environment* **218**, 66-72, doi:10.1016/j.agee.2015.11.014 (2016).

2 Aerts, R., Cornelissen, J. H. C. & Dorrepaal, E. Plant performance in a warmer world: general responses of plants from cold, northern biomes and the importance of winter and spring events. *Plant Ecology* **182**, 65-77, doi:10.1007/s11258-005-9031-1 (2006).

3 Albert, K. R. *et al.* Interactive effects of elevated CO_2, warming, and drought on photosynthesis of Deschampsia flexuosa in a temperate heath ecosystem. *J Exp Bot* **62**, 4253-4266, doi:10.1093/jxb/err133 (2011).

4 Albert, K. R. *et al.* Effects of elevated CO_2, warming and drought episodes on plant carbon uptake in a temperate heath ecosystem are controlled by soil water status. *Plant Cell Environ* **34**, 1207-1222, doi:10.1111/j.1365-3040.2011.02320.x (2011).

5 Allison, S. D., McGuire, K. L. & Treseder, K. K. Resistance of microbial and soil properties to warming treatment seven years after boreal fire. *Soil Biology and Biochemistry* **42**, 1872-1878, doi:10.1016/j.soilbio.2010.07.011 (2010).

6 Allison, S. D. & Treseder, K. K. Warming and drying suppress microbial activity and carbon cycling in boreal forest soils. *Global Change Biology* **14**, 2898-2909, doi:10.1111/j.1365-2486.2008.01716.x (2008).

7 Almagro, M., Maestre, F. T., Martínez-López, J., Valencia, E. & Rey, A. Climate change may reduce litter decomposition while enhancing the contribution of photodegradation in dry perennial Mediterranean grasslands. *Soil Biology and Biochemistry* **90**, 214-223, doi:10.1016/j.soilbio.2015.08.006 (2015).

8 Ambebe, T. F. & Dang, Q. L. Low moisture availability inhibits the enhancing effect of increased soil temperature on net photosynthesis of white birch (*Betula papyrifera*) seedlings grown under ambient and elevated carbon dioxide concentrations. *Tree Physiol* **29**, 1341-1348, doi:10.1093/treephys/tpp079 (2009).

9 Ambebe, T. F., Dang, Q.-L. & Marfo, J. Low soil temperature reduces the positive effects of high nutrient supply on the growth and biomass of white birch seedlings in ambient and elevated carbon dioxide concentrations. *Botany* **87**, 905-912, doi:10.1139/b09-060 (2009).

10 An, J. *et al.* Physiological and growth responses to experimental warming in first-year seedlings of deciduous tree species. *Turkish Journal of Agriculture and Forestry* **41**, 175-182, doi:10.3906/tar-1611-106 (2017).

11 Anadon-Rosell, A. *et al.* Four years of experimental warming do not modify the interaction between subalpine shrub species. *Oecologia* **183**, 1167-1181, doi:10.1007/s00442-017-3830-7 (2017).

12 Andresen, L. C., Michelsen, A., Ambus, P. & Beier, C. Belowground heathland responses after 2 years of combined warming, elevated CO_2 and summer drought. *Biogeochemistry* **101**, 27-42, doi:10.1007/s10533-010-9489-3 (2010).

13 Andresen, L. C. *et al.* Plant nutrient mobilization in temperate heathland responds to elevated CO_2, temperature and drought. *Plant and Soil* **328**, 381-396, doi:10.1007/s11104-009-0118-7 (2010).

14 Arnold, S. S., Fernandez, I. J., Rustad, L. E. & Zibilske, L. M. Microbial response of an acid forest soil to experimental soil warming. *Biology and Fertility of Soils* **30**, 239-244, doi:10.1007/s003740050614 (1999).

15 Bai, W. *et al.* Increased temperature and precipitation interact to affect root production, mortality, and turnover in a temperate steppe: implications for ecosystem C cycling. *Global Change Biology* **16**, 1306-1316, doi:10.1111/j.1365-2486.2009.02019.x (2010).

16 Bamminger, C., Poll, C. & Marhan, S. Offsetting global warming-induced elevated greenhouse gas emissions

from an arable soil by biochar application. *Glob Chang Biol* **24**, e318-e334, doi:10.1111/gcb.13871 (2018).

17 Bamminger, C. *et al.* Short-term response of soil microorganisms to biochar addition in a temperate agroecosystem under soil warming. *Agriculture, Ecosystems & Environment* **233**, 308-317, doi:10.1016/j.agee.2016.09.016 (2016).

18 Bannayan, M., Tojo Soler, C. M., Garcia y. Garcia, A., Guerra, L. C. & Hoogenboom, G. Interactive effects of elevated [CO_2] and temperature on growth and development of a short- and long-season peanut cultivar. *Climatic Change* **93**, 389-406, doi:10.1007/s10584-008-9510-1 (2008).

19 Barnard, R. *et al.* Several components of global change alter nitrifying and denitrifying activities in an annual grassland. *Functional Ecology* **20**, 557-564, doi:10.1111/j.1365-2435.2006.01146.x (2006).

20 Bassirirad, H., Tissue, D. T., Reynolds, J. F. & Chapin, F. S. Response of *Eriophorum vaginatum* to CO_2 enrichment at different soil temperatures: Effects on growth, root respiration and PO_4^{3-} uptake kinetics. *New Phytologist* **133**, 423-430, doi:10.1111/j.1469-8137.1996.tb01909.x (1996).

21 Belay-Tedla, A., Zhou, X., Su, B., Wan, S. & Luo, Y. Labile, recalcitrant, and microbial carbon and nitrogen pools of a tallgrass prairie soil in the US Great Plains subjected to experimental warming and clipping. *Soil Biology and Biochemistry* **41**, 110-116, doi:10.1016/j.soilbio.2008.10.003 (2009).

22 Bergner, B., Johnstone, J. & Treseder, K. K. Experimental warming and burn severity alter soil CO_2 flux and soil functional groups in a recently burned boreal forest. *Global Change Biology* **10**, 1996-2004, doi:10.1111/j.1365-2486.2004.00868.x (2004).

23 Biasi, C. *et al.* Initial effects of experimental warming on carbon exchange rates, plant growth and microbial dynamics of a lichen-rich dwarf shrub tundra in Siberia. *Plant and Soil* **307**, 191-205, doi:10.1007/s11104-008-9596-2 (2008).

24 Birgander, J., Rousk, J. & Olsson, P. A. Warmer winters increase the rhizosphere carbon flow to mycorrhizal fungi more than to other microorganisms in a temperate grassland. *Global Change Biology* **23**, 5372-5382, doi:10.1111/gcb.13803 (2017).

25 Bjork, R. G., Majdi, H., Klemedtsson, L., Lewis-Jonsson, L. & Molau, U. Long-term warming effects on root morphology, root mass distribution, and microbial activity in two dry tundra plant communities in northern Sweden. *New Phytol* **176**, 862-873, doi:10.1111/j.1469-8137.2007.02231.x (2007).

26 Black, C. K., Davis, S. C., Hudiburg, T. W., Bernacchi, C. J. & DeLucia, E. H. Elevated CO_2 and temperature increase soil C losses from a soybean-maize ecosystem. *Glob Chang Biol* **23**, 435-445, doi:10.1111/gcb.13378 (2017).

27 Bloor, J. M. G., Pichon, P., Falcimagne, R., Leadley, P. & Soussana, J.-F. Effects of warming, summer drought, and CO_2 enrichment on aboveground biomass production, flowering phenology, and community structure in an upland grassland ecosystem. *Ecosystems* **13**, 888-900, doi:10.1007/s10021-010-9363-0 (2010).

28 Boelman, N. T. *et al.* Response of NDVI, biomass, and ecosystem gas exchange to long-term warming and fertilization in wet sedge tundra. *Oecologia* **135**, 414-421, doi:10.1007/s00442-003-1198-3 (2003).

29 Bokhorst, S., Bjerke, J. W., Melillo, J., Callaghan, T. V. & Phoenix, G. K. Impacts of extreme winter warming events on litter decomposition in a sub-Arctic heathland. *Soil Biology and Biochemistry* **42**, 611-617, doi:10.1016/j.soilbio.2009.12.011 (2010).

30 Bokhorst, S., Huiskes, A., Convey, P. & Aerts, R. Climate change effects on organic matter decomposition rates in ecosystems from the Maritime Antarctic and Falkland Islands. *Global Change Biology* **13**, 2642-2653, doi:10.1111/j.1365-2486.2007.01468.x (2007).

31 Bracho, R. *et al.* Temperature sensitivity of organic matter decomposition of permafrost-region soils during laboratory incubations. *Soil Biology and Biochemistry* **97**, 1-14, doi:10.1016/j.soilbio.2016.02.008 (2016).

32 Bradford, M. A. *et al.* Thermal adaptation of soil microbial respiration to elevated temperature. *Ecol Lett* **11**, 1316-1327, doi:10.1111/j.1461-0248.2008.01251.x (2008).

33 Briones, M. J. I., Ostle, N. J., McNamara, N. P. & Poskitt, J. Functional shifts of grassland soil communities in response to soil warming. *Soil Biology and Biochemistry* **41**, 315-322, doi:10.1016/j.soilbio.2008.11.003 (2009).

34 Bronson, D. R., Gower, S. T., Tanner, M., Linder, S. & Van Herk, I. Response of soil surface CO_2 flux in a boreal forest to ecosystem warming. *Global Change Biology* **14**, 856-867, doi:10.1111/j.1365-2486.2007.01508.x (2007).

35 Broughton, K. J. *et al.* Warming alters the positive impact of elevated CO_2 concentration on cotton growth and physiology during soil water deficit. *Funct Plant Biol* **44**, 267-278, doi:10.1071/FP16189 (2017).

36 Cai, C. *et al.* Responses of wheat and rice to factorial combinations of ambient and elevated CO_2 and temperature in FACE experiments. *Glob Chang Biol* **22**, 856-874, doi:10.1111/gcb.13065 (2016).

37 Cao, J. & Ruan, H. Responses of the submerged macrophyte Vallisneria natans to elevated CO_2 and temperature. *Aquatic Biology* **23**, 119-127, doi:10.3354/ab00605 (2015).

38 Carrera, N., Barreal, M. E., Rodeiro, J. & Briones, M. J. I. Interactive effects of temperature, soil moisture and enchytraeid activities on C losses from a peatland soil. *Pedobiologia* **54**, 291-299, doi:10.1016/j.pedobi.2011.07.002 (2011).

39 Carrillo, Y., Dijkstra, F. A., Pendall, E., Morgan, J. A. & Blumenthal, D. M. Controls over soil nitrogen pools in a semiarid grassland under elevated CO_2 and warming. *Ecosystems* **15**, 761-774, doi:10.1007/s10021-012-9544-0 (2012).

40 Carrillo, Y., Pendall, E., Dijkstra, F. A., Morgan, J. A. & Newcomb, J. M. Response of soil organic matter pools to elevated CO_2 and warming in a semi-arid grassland. *Plant and Soil* **347**, 339-350, doi:10.1007/s11104-011-0853-4 (2011).

41 Carter, E. B., Theodorou, M. K. & Morris, P. Responses of *Lotus corniculatus* to environmental change. 2. Effect of elevated CO_2, temperature and drought on tissue digestion in relation to condensed tannin and carbohydrate accumulation. *Journal of the Science of Food and Agriculture* **79**, 1431-1440, doi:10.1002/(sici)1097-0010(199908)79:11<1431::Aid-jsfa384>3.0.Co;2-4 (1999).

42 Chang, R., Wang, G., Yang, Y. & Chen, X. Experimental warming increased soil nitrogen sink in the Tibetan permafrost. *Journal of Geophysical Research: Biogeosciences* **122**, 1870-1879, doi:10.1002/2017jg003827 (2017).

43 Chapin, F. S., III, Shaver, G. R., Giblin, A. E., Nadelhoffer, K. J. & Laundre, J. A. Response of arctic tundra to experimental and observed changes in climate. *Ecology (Washington D C)* **76**, 694-711 (1995).

44 Charles, H. & Dukes, J. S. Effects of warming and altered precipitation on plant and nutrient dynamics of a New England salt marsh. *Ecological Applications* **19**, 1758-1773, doi:10.1890/08-0172.1 (2009).

45 Chen, H. *et al.* Chemistry and long-term decomposition of roots of Douglas-fir grown under elevated atmospheric carbon dioxide and warming conditions. *J Environ Qual* **37**, 1327-1336, doi:10.2134/jeq2007.0266 (2008).

46 Chen, J. *et al.* Differential responses of ecosystem respiration components to experimental warming in a meadow grassland on the Tibetan Plateau. *Agricultural and Forest Meteorology* **220**, 21-29, doi:10.1016/j.agrformet.2016.01.010 (2016).

47 Chen, X. *et al.* Effects of warming and nitrogen fertilization on GHG flux in the permafrost region of an alpine meadow. *Atmospheric Environment* **157**, 111-124, doi:10.1016/j.atmosenv.2017.03.024 (2017).

48 Chen, X. *et al.* Effects of warming and nitrogen fertilization on GHG flux in an alpine swamp meadow of a permafrost region. *Sci Total Environ* **601-602**, 1389-1399, doi:10.1016/j.scitotenv.2017.06.028 (2017).

49 Chen, Z., Zhang, J., Xiong, Z., Pan, G. & Müller, C. Enhanced gross nitrogen transformation rates and nitrogen supply in paddy field under elevated atmospheric carbon dioxide and temperature. *Soil Biology and Biochemistry* **94**, 80-87, doi:10.1016/j.soilbio.2015.11.025 (2016).
50 Cheng, H., Ren, W., Ding, L., Liu, Z. & Fang, C. Responses of a rice–wheat rotation agroecosystem to experimental warming. *Ecological Research* **28**, 959-967, doi:10.1007/s11284-013-1078-1 (2013).
51 Cheng, X. *et al.* Experimental warming and clipping altered litter carbon and nitrogen dynamics in a tallgrass prairie. *Agriculture, Ecosystems & Environment* **138**, 206-213, doi:10.1016/j.agee.2010.04.019 (2010).
52 Chivers, M. R., Turetsky, M. R., Waddington, J. M., Harden, J. W. & McGuire, A. D. Effects of experimental water table and temperature manipulations on ecosystem CO_2 fluxes in an Alaskan rich fen. *Ecosystems* **12**, 1329-1342, doi:10.1007/s10021-009-9292-y (2009).
53 Choi, E.-Y., Seo, T.-C., Lee, S.-G., Cho, I.-H. & Stangoulis, J. Growth and physiological responses of Chinese cabbage and radish to long-term exposure to elevated carbon dioxide and temperature. *Horticulture, Environment, and Biotechnology* **52**, 376-386, doi:10.1007/s13580-011-0012-0 (2011).
54 Christensen, T. R., Michelsen, A., Jonasson, S. & Schmidt, I. K. Carbon dioxide and methane exchange of a subarctic heath in response to climate change related environmental manipulations. *Oikos* **79**, 34-44, doi:10.2307/3546087 (1997).
55 Christiansen, C. T. *et al.* Enhanced summer warming reduces fungal decomposer diversity and litter mass loss more strongly in dry than in wet tundra. *Glob Chang Biol* **23**, 406-420, doi:10.1111/gcb.13362 (2017).
56 Clausen, S. K. *et al.* Effects of single and multifactor treatments with elevated temperature, CO_2 and ozone on *Oilseed Rape* and barley. *Journal of Agronomy and Crop Science* **197**, 442-453, doi:10.1111/j.1439-037X.2011.00478.x (2011).
57 Clemmensen, K. E. & Michelsen, A. Integrated long-term responses of an arctic–alpine willow and associated ectomycorrhizal fungi to an altered environment. *Canadian Journal of Botany* **84**, 831-843, doi:10.1139/b06-039 (2006).
58 Coldren, G. A. *et al.* Chronic warming stimulates growth of marsh grasses more than mangroves in a coastal wetland ecotone. *Ecology* **97**, 3167-3175, doi:10.1002/ecy.1539 (2016).
59 Comstedt, D. *et al.* Effects of elevated atmospheric carbon dioxide and temperature on soil respiration in a boreal forest using δ13C as a labeling tool. *Ecosystems* **9**, 1266-1277, doi:10.1007/s10021-006-0110-5 (2007).
60 Darrouzet-Nardi, A., Reed, S. C., Grote, E. E. & Belnap, J. Observations of net soil exchange of CO_2 in a dryland show experimental warming increases carbon losses in biocrust soils. *Biogeochemistry* **126**, 363-378, doi:10.1007/s10533-015-0163-7 (2015).
61 Day, T. A., Ruhland, C. T. & Xiong, F. S. Warming increases aboveground plant biomass and C stocks in vascular-plant-dominated Antarctic tundra. *Global Change Biology* **14**, 1827-1843, doi:10.1111/j.1365-2486.2008.01623.x (2008).
62 De Boeck, H. J. *et al.* How do climate warming and species richness affect CO_2 fluxes in experimental grasslands? *New Phytol* **175**, 512-522, doi:10.1111/j.1469-8137.2007.02122.x (2007).
63 de Dato, G. D., De Angelis, P., Sirca, C. & Beier, C. Impact of drought and increasing temperatures on soil CO_2 emissions in a Mediterranean shrubland (gariga). *Plant and Soil* **327**, 153-166, doi:10.1007/s11104-009-0041-y (2009).
64 De Valpine, P. & Harte, J. Plant responses to experimental warming in a montane meadow. *Ecology* **82**, 637-648, doi:10.1890/0012-9658(2001)082[0637:Prtewi]2.0.Co;2 (2001).
65 Deane-Coe, K. K. *et al.* Experimental warming alters productivity and isotopic signatures of tundra mosses. *Ecosystems* **18**, 1070-1082, doi:10.1007/s10021-015-9884-7 (2015).

66 Delarue, F. *et al.* Indirect effects of experimental warming on dissolved organic carbon content in subsurface peat. *Journal of Soils and Sediments* **14**, 1800-1805, doi:10.1007/s11368-014-0945-x (2014).
67 Dijkstra, F. A. *et al.* Contrasting effects of elevated CO_2 and warming on nitrogen cycling in a semiarid grassland. *New Phytol* **187**, 426-437, doi:10.1111/j.1469-8137.2010.03293.x (2010).
68 D'Imperio, L., Nielsen, C. S., Westergaard-Nielsen, A., Michelsen, A. & Elberling, B. Methane oxidation in contrasting soil types: responses to experimental warming with implication for landscape-integrated CH_4 budget. *Glob Chang Biol* **23**, 966-976, doi:10.1111/gcb.13400 (2017).
69 Domínguez, M. T., Holthof, E., Smith, A. R., Koller, E. & Emmett, B. A. Contrasting response of summer soil respiration and enzyme activities to long-term warming and drought in a wet shrubland (NE Wales, UK). *Applied Soil Ecology* **110**, 151-155, doi:10.1016/j.apsoil.2016.11.003 (2017).
70 Dukes, J. S. *et al.* Responses of grassland production to single and multiple global environmental changes. *PLoS Biol* **3**, e319, doi:10.1371/journal.pbio.0030319 (2005).
71 Dunne, J. A., Saleska, S. R., Fischer, M. L. & Harte, J. Integrating experimental and gradient methods in ecological climate change research. *Ecology* **85**, 904-916, doi:10.1890/03-8003 (2004).
72 Edwards, E. J., Benham, D. G., Marland, L. A. & Fitter, A. H. Root production is determined by radiation flux in a temperate grassland community. *Global Change Biology* **10**, 209-227, doi:10.1111/j.1365-2486.2004.00729.x (2004).
73 Edwards, E. J., Unwin, D., Kilmister, R. & Treeby, M. Multi-seasonal effects of warming and elevated CO_2 on the physiology, growth and production of mature, field grown, Shiraz grapevines. *Oeno One* **51**, 127-132, doi:10.20870/oeno-one.2016.0.0.1586 (2017).
74 Edwards, N. T. & Norby, R. J. Below-ground respiratory responses of sugar maple and red maple saplings to atmospheric CO_2 enrichment and elevated air temperature. *Plant and Soil* **206**, 85-97 (1999).
75 Emmett, B. A. *et al.* The response of soil processes to climate change: Results from manipulation studies of shrublands across an environmental gradient. *Ecosystems* **7**, doi:10.1007/s10021-004-0220-x (2004).
76 Erice, G., Irigoyen, J. J., Perez, P., Martinez-Carrasco, R. & Sanchez-Diaz, M. Effect of elevated CO_2, temperature and drought on photosynthesis of nodulated alfalfa during a cutting regrowth cycle. *Physiologia Plantarum* **126**, 458-468, doi:10.1111/j.1399-3054.2006.00599.x (2006).
77 Fang, C. *et al.* Seasonal responses of soil respiration to warming and nitrogen addition in a semi-arid alfalfa-pasture of the Loess Plateau, China. *Sci Total Environ* **590-591**, 729-738, doi:10.1016/j.scitotenv.2017.03.034 (2017).
78 Farfan-Vignolo, E. R. & Asard, H. Effect of elevated CO_2 and temperature on the oxidative stress response to drought in *Lolium perenne* L. and *Medicago sativa* L. *Plant Physiol Biochem* **59**, 55-62, doi:10.1016/j.plaphy.2012.06.014 (2012).
79 Fenner, N. *et al.* Interactions between elevated CO_2 and warming could amplify DOC exports from peatland catchments. *Environmental Science & Technology* **41**, 3146-3152, doi:10.1021/es061765v (2007).
80 Fernandez, C. W. *et al.* Ectomycorrhizal fungal response to warming is linked to poor host performance at the boreal-temperate ecotone. *Glob Chang Biol* **23**, 1598-1609, doi:10.1111/gcb.13510 (2017).
81 Ferreira, V., Chauvet, E. & Canhoto, C. Effects of experimental warming, litter species, and presence of macroinvertebrates on litter decomposition and associated decomposers in a temperate mountain stream. *Canadian Journal of Fisheries and Aquatic Sciences* **72**, 206-216, doi:10.1139/cjfas-2014-0119 (2015).
82 Fu, G. *et al.* Experimental warming does not enhance gross primary production and above-ground biomass in the alpine meadow of Tibet. *Journal of Applied Remote Sensing* **7**, doi:10.1117/1.Jrs.7.073505 (2013).
83 Fujimura, K. E., Egger, K. N. & Henry, G. H. The effect of experimental warming on the root-associated fungal community of *Salix arctica*. *ISME J* **2**, 105-114, doi:10.1038/ismej.2007.89 (2008).

84 FuSun, S. H. I., Ning, W. U. & Peng, L. U. O. Effect of temperature enhancement on community structure and biomass of subaipine meadow in Northwestern Sichuan. *Acta Ecologica Sinica* **28**, 5286-5293 (2008).

85 Ganjurjav, H. *et al.* Differential response of alpine steppe and alpine meadow to climate warming in the central Qinghai–Tibetan Plateau. *Agricultural and Forest Meteorology* **223**, 233-240, doi:10.1016/j.agrformet.2016.03.017 (2016).

86 Garten, C. T., Classen, A. T. & Norby, R. J. Soil moisture surpasses elevated CO_2 and temperature as a control on soil carbon dynamics in a multi-factor climate change experiment. *Plant and Soil* **319**, 85-94, doi:10.1007/s11104-008-9851-6 (2008).

87 Ge, Z. M. *et al.* Responses of leaf photosynthesis, pigments and chlorophyll fluorescence within canopy position in a boreal grass (*Phalaris arundinacea* L.) to elevated temperature and CO_2 under varying water regimes. *Photosynthetica* **49**, 172-184, doi:10.1007/s11099-011-0029-8 (2011).

88 Ge, Z.-M. *et al.* Measured and modeled biomass growth in relation to photosynthesis acclimation of a bioenergy crop (Reed canary grass) under elevated temperature, CO_2 enrichment and different water regimes. *Biomass and Bioenergy* **46**, 251-262, doi:10.1016/j.biombioe.2012.08.019 (2012).

89 Gong, S. *et al.* Response of soil enzyme activity to warming and nitrogen addition in a meadow steppe. *Soil Research* **53**, doi:10.1071/sr14140 (2015).

90 Gonzalez-Meler, M. A., Silva, L. B., Dias-De-Oliveira, E., Flower, C. E. & Martinez, C. A. Experimental air warming of a *Stylosanthes capitata* Vogel dominated tropical pasture affects soil respiration and nitrogen dynamics. *Front Plant Sci* **8**, 46, doi:10.3389/fpls.2017.00046 (2017).

91 Gorissen, A. *et al.* Climate Change Affects Carbon Allocation to the Soil in Shrublands. *Ecosystems* **7**, doi:10.1007/s10021-004-0218-4 (2004).

92 Graham, S. L. *et al.* Effects of soil warming and nitrogen addition on soil respiration in a New Zealand tussock grassland. *PLoS One* **9**, e91204, doi:10.1371/journal.pone.0091204 (2014).

93 Grogan, P. & Chapin Iii, F. S. Initial effects of experimental warming on above- and belowground components of net ecosystem CO_2 exchange in arctic tundra. *Oecologia* **125**, 512-520, doi:10.1007/s004420000490 (2000).

94 Guoju, X. *et al.* Warming influences the yield and water use efficiency of winter wheat in the semiarid regions of Northwest China. *Field Crops Research* **199**, 129-135, doi:10.1016/j.fcr.2016.09.023 (2016).

95 Hagedorn, F. *et al.* Short-term responses of ecosystem carbon fluxes to experimental soil warming at the Swiss alpine treeline. *Biogeochemistry* **97**, 7-19, doi:10.1007/s10533-009-9297-9 (2009).

96 Hakala, K. Growth and yield potential of spring wheat in a simulated changed climate with increased CO_2 and higher temperature. *European Journal of Agronomy* **9**, 41-52, doi:10.1016/s1161-0301(98)00025-2 (1998).

97 Han, S. *et al.* Species-specific growth and photosynthetic responses of first-year seedlings of four coniferous species to open-field experimental warming. *Turkish Journal of Agriculture and Forestry* **39**, 342-349, doi:10.3906/tar-1408-117 (2015).

98 Harte, J., Saleska, S. R. & Levy, C. Convergent ecosystem responses to 23-year ambient and manipulated warming link advancing snowmelt and shrub encroachment to transient and long-term climate-soil carbon feedback. *Glob Chang Biol* **21**, 2349-2356, doi:10.1111/gcb.12831 (2015).

99 Hartley, A. E., Neill, C., Melillo, J. M., Crabtree, R. & Bowles, F. P. Plant performance and soil nitrogen mineralization in response to simulated climate change in subarctic dwarf shrub heath. *Oikos* **86**, 331-343, doi:10.2307/3546450 (1999).

100 Hartley, I. P., Heinemeyer, A., Evans, S. P. & Ineson, P. The effect of soil warming on bulk soil vs. rhizosphere respiration. *Global Change Biology* **13**, 2654-2667, doi:10.1111/j.1365-2486.2007.01454.x (2007).

101 Hartley, I. P., Heinemeyer, A. & Ineson, P. Effects of three years of soil warming and shading on the rate of soil respiration: substrate availability and not thermal acclimation mediates observed response. *Global Change Biology* **13**, 1761-1770, doi:10.1111/j.1365-2486.2007.01373.x (2007).

102 Haugwitz, M. S. *et al.* Soil microorganisms respond to five years of climate change manipulations and elevated atmospheric CO_2 in a temperate heath ecosystem. *Plant and Soil* **374**, 211-222, doi:10.1007/s11104-013-1855-1 (2013).

103 Hayden, H. L. *et al.* Changes in the microbial community structure of bacteria, archaea and fungi in response to elevated CO_2 and warming in an Australian native grassland soil. *Environ Microbiol* **14**, 3081-3096, doi:10.1111/j.1462-2920.2012.02855.x (2012).

104 He, J., Austin, P. T. & Lee, S. K. Effects of elevated root zone CO_2 and air temperature on photosynthetic gas exchange, nitrate uptake, and total reduced nitrogen content in aeroponically grown lettuce plants. *J Exp Bot* **61**, 3959-3969, doi:10.1093/jxb/erq207 (2010).

105 Henry, H. A. L., Brizgys, K. & Field, C. B. Litter decomposition in a california annual grassland: Interactions between photodegradation and litter layer thickness. *Ecosystems* **11**, 545-554, doi:10.1007/s10021-008-9141-4 (2008).

106 Henry, H. A. L., Hutchison, J. S., Kim, M. K. & McWhirter, B. D. Context matters for warming: Interannual variation in grass biomass responses to 7 years of warming and N addition. *Ecosystems* **18**, 103-114, doi:10.1007/s10021-014-9816-y (2014).

107 Heskel, M. A. *et al.* Thermal acclimation of shoot respiration in an Arctic woody plant species subjected to 22 years of warming and altered nutrient supply. *Glob Chang Biol* **20**, 2618-2630, doi:10.1111/gcb.12544 (2014).

108 Hicks Pries, C. E. *et al.* Decadal warming causes a consistent and persistent shift from heterotrophic to autotrophic respiration in contrasting permafrost ecosystems. *Glob Chang Biol* **21**, 4508-4519, doi:10.1111/gcb.13032 (2015).

109 Hill, P. W. *et al.* Living roots magnify the response of soil organic carbon decomposition to temperature in temperate grassland. *Global Change Biology* **21**, 1368-1375, doi:10.1111/gcb.12784 (2015).

110 Hines, J., Reyes, M. & Gessner, M. O. Density constrains cascading consequences of warming and nitrogen from invertebrate growth to litter decomposition. *Ecology* **97**, 1635-1642, doi:10.1002/ecy.1447 (2016).

111 Hobbie, S. E. & Chapin, F. S. Response of tundra plant biomass, aboveground production, nitrogen, and CO_2 flux to experimental warming. *Ecology* **79**, 1526-1544 (1998).

112 Hou, R., Ouyang, Z., Maxim, D., Wilson, G. & Kuzyakov, Y. Lasting effect of soil warming on organic matter decomposition depends on tillage practices. *Soil Biology and Biochemistry* **95**, 243-249, doi:10.1016/j.soilbio.2015.12.008 (2016).

113 Hou, Y., Qu, J., Luo, Z., Zhang, C. & Wang, K. Morphological mechanism of growth response in treeline species Minjiang fir to elevated CO_2 and temperature. *Silva Fennica* **45**, 181-195, doi:10.14214/sf.41 (2011).

114 Hovenden, M. J. *et al.* Warming and elevated CO_2 affect the relationship between seed mass, germinability and seedling growth in *Austrodanthonia caespitosa*, a dominant Australian grass. *Global Change Biology* **14**, 1633-1641, doi:10.1111/j.1365-2486.2008.01597.x (2008).

115 Hu, H.-W. *et al.* Effects of climate warming and elevated CO_2 on autotrophic nitrification and nitrifiers in dryland ecosystems. *Soil Biology and Biochemistry* **92**, 1-15, doi:10.1016/j.soilbio.2015.09.008 (2016).

116 Huang, G., Rymer, P. D., Duan, H., Smith, R. A. & Tissue, D. T. Elevated temperature is more effective than elevated [CO_2] in exposing genotypic variation in *Telopea speciosissima* growth plasticity: Implications for woody plant populations under climate change. *Glob Chang Biol* **21**, 3800-3813, doi:10.1111/gcb.12990 (2015).

117 Huang, S., Jia, X., Zhao, Y., Chang, Y. & Bai, B. Response of *Robinia pseudoacacia* L. rhizosphere microenvironment to Cd and Pb contamination and elevated temperature. *Applied Soil Ecology* **108**, 269-277, doi:10.1016/j.apsoil.2016.09.002 (2016).

118 Huttunen, L. *et al.* Effects of elevated temperature, elevated CO_2 and fertilization on quality and subsequent decomposition of silver birch leaf litter. *Soil Biology and Biochemistry* **41**, 2414-2421, doi:10.1016/j.soilbio.2009.08.014 (2009).

119 Illeris, L. *et al.* Growing-season carbon dioxide flux in a dry subarctic heath: Responses to long-term manipulations. *Arctic Antarctic and Alpine Research* **36**, 456-463, doi:10.1657/1523-0430(2004)036[0456:Gcdfia]2.0.Co;2 (2004).

120 Jansen-Willems, A. B., Lanigan, G. J., Grunhage, L. & Muller, C. Carbon cycling in temperate grassland under elevated temperature. *Ecol Evol* **6**, 7856-7868, doi:10.1002/ece3.2210 (2016).

121 Jarvi, M. P. & Burton, A. J. Acclimation and soil moisture constrain sugar maple root respiration in experimentally warmed soil. *Tree Physiol* **33**, 949-959, doi:10.1093/treephys/tpt068 (2013).

122 Jassey, V. E. *et al.* An unexpected role for mixotrophs in the response of peatland carbon cycling to climate warming. *Sci Rep* **5**, 16931, doi:10.1038/srep16931 (2015).

123 Jiang, L. *et al.* Effects of warming and grazing on dissolved organic nitrogen in a Tibetan alpine meadow ecosystem. *Soil and Tillage Research* **158**, 156-164, doi:10.1016/j.still.2015.12.012 (2016).

124 Johnson, L. C. *et al.* Plant carbon-nutrient interactions control CO_2 exchange in Alaskan wet sedge tundra ecosystems. *Ecology* **81**, 453-469, doi:10.1890/0012-9658(2000)081[0453:Pcnicc]2.0.Co;2 (2000).

125 Jonasson, S., Michelsen, A., Schmidt, I. K. & Nielsen, E. V. Responses in microbes and plants to changed temperature, nutrient, and light regimes in the arctic. *Ecology* **80**, 1828-1843, doi:10.2307/176661 (1999).

126 Jónsdóttir, I. S., Khitun, O. & Stenström, A. Biomass and nutrient responses of a clonal tundra sedge to climate warming. *Canadian Journal of Botany* **83**, 1608-1621, doi:10.1139/b05-129 (2005).

127 Kandeler, E. *et al.* The response of soil microorganisms and roots to elevated CO_2 and temperature in a terrestrial model ecosystem. *Plant and Soil* **202**, 251-262, doi:10.1023/a:1004309623256 (1998).

128 Kardol, P. *et al.* Climate change effects on plant biomass alter dominance patterns and community evenness in an experimental old-field ecosystem. *Global Change Biology* **16**, 2676-2687, doi:10.1111/j.1365-2486.2010.02162.x (2010).

129 Kim, H.-Y. *et al.* Dry matter and nitrogen accumulation and partitioning in rice (*Oryza sativa* L.) exposed to experimental warming with elevated CO_2. *Plant and Soil* **342**, 59-71, doi:10.1007/s11104-010-0665-y (2010).

130 King, J. S., Thomas, R. B. & Strain, B. R. Growth and carbon accumulation in root systems of *Pinus taeda* and *Pinus ponderosa* seedlings as affected by varying CO_2, temperature and nitrogen. *Tree Physiology* **16**, 635-642 (1996).

131 Kivimaenpaa, M. *et al.* Warming and elevated ozone differently modify needle anatomy of Norway spruce (*Picea abies*) and Scots pine (*Pinus sylvestris*). *Canadian Journal of Forest Research* **47**, 488-499, doi:10.1139/cjfr-2016-0406 (2017).

132 Kreyling, J. *et al.* Nitrogen leaching is enhanced after a winter warm spell but mainly controlled by vegetation composition in temperate zone mesocosms. *Plant and Soil* **396**, 85-96, doi:10.1007/s11104-015-2587-1 (2015).

133 Kuster, T. M., Schleppi, P., Hu, B., Schulin, R. & Gunthardt-Goerg, M. S. Nitrogen dynamics in oak model ecosystems subjected to air warming and drought on two different soils. *Plant Biol (Stuttg)* **15 Suppl 1**, 220-229, doi:10.1111/j.1438-8677.2012.00686.x (2013).

134 Lee, J.-S. Combined effect of elevated CO_2 and temperature on the growth and phenology of two annual C3

and C4 weedy species. *Agriculture, Ecosystems & Environment* **140**, 484-491, doi:10.1016/j.agee.2011.01.013 (2011).

135 Lewis, J. D., Lucash, M., Olszyk, D. M. & Tingey, D. T. Relationships between needle nitrogen concentration and photosynthetic responses of Douglas-fir seedlings to elevated CO_2 and temperature. *New Phytologist* **162**, 355-364, doi:10.1111/j.1469-8137.2004.01036.x (2004).

136 Li, D., Zhou, X., Wu, L., Zhou, J. & Luo, Y. Contrasting responses of heterotrophic and autotrophic respiration to experimental warming in a winter annual-dominated prairie. *Glob Chang Biol* **19**, 3553-3564, doi:10.1111/gcb.12273 (2013).

137 Li, F. *et al.* Warming effects on permafrost ecosystem carbon fluxes associated with plant nutrients. *Ecology* **98**, 2851-2859, doi:10.1002/ecy.1975 (2017).

138 Li, G., Kim, S., Han, S., Chang, H. & Son, Y. Effect of soil moisture on the response of soil respiration to open-field experimental warming and precipitation manipulation. *Forests* **8**, doi:10.3390/f8030056 (2017).

139 Li, Y. *et al.* Effects of warming on ectomycorrhizal colonization and nitrogen nutrition of *Picea asperata* seedlings grown in two contrasting forest ecosystems. *Sci Rep* **5**, 17546, doi:10.1038/srep17546 (2015).

140 Liancourt, P. *et al.* Leaf-trait plasticity and species vulnerability to climate change in a Mongolian steppe. *Glob Chang Biol* **21**, 3489-3498, doi:10.1111/gcb.12934 (2015).

141 Lin, G. H., Ehleringer, J. R., Rygiewicz, P. T., Johnson, M. G. & Tingey, D. T. Elevated CO_2 and temperature impacts on different components of soil CO_2 efflux in Douglas-fir terracosms. *Global Change Biology* **5**, 157-168, doi:10.1046/j.1365-2486.1999.00211.x (1999).

142 Lin, G. H., Rygiewicz, P. T., Ehleringer, J. R., Johnson, M. G. & Tingey, D. T. Time-dependent responses of soil CO_2 efflux components to elevated atmospheric CO_2 and temperature in experimental forest mesocosms. *Plant and Soil* **229**, 259-270, doi:10.1023/a:1004883221036 (2001).

143 Lin, X. *et al.* Experimental warming increases seasonal methane uptake in an alpine meadow on the Tibetan Plateau. *Ecosystems* **18**, 274-286, doi:10.1007/s10021-014-9828-7 (2014).

144 Lin, X. *et al.* Response of ecosystem respiration to warming and grazing during the growing seasons in the alpine meadow on the Tibetan plateau. *Agricultural and Forest Meteorology* **151**, 792-802, doi:10.1016/j.agrformet.2011.01.009 (2011).

145 Litvak, M. E., Constable, J. V. & Monson, R. K. Supply and demand processes as controls over needle monoterpene synthesis and concentration in Douglas fir [*Pseudotsuga menziesii* (Mirb.) Franco]. *Oecologia* **132**, 382-391, doi:10.1007/s00442-002-0964-y (2002).

146 Liu, C. & Zou, D. Do increased temperature and CO_2 levels affect the growth, photosynthesis, and respiration of the marine macroalga *Pyropia haitanensis* (Rhodophyta)? An experimental study. *Hydrobiologia* **745**, 285-296, doi:10.1007/s10750-014-2113-0 (2014).

147 Liu, D. *et al.* Physiological adjustments of a Mediterranean shrub to long-term experimental warming and drought treatments. *Plant Sci* **252**, 53-61, doi:10.1016/j.plantsci.2016.07.004 (2016).

148 Liu, L., Hu, C., Olesen, J. E., Ju, Z. & Zhang, X. Effect of warming and nitrogen addition on evapotranspiration and water use efficiency in a wheat-soybean/fallow rotation from 2010 to 2014. *Climatic Change* **139**, 565-578, doi:10.1007/s10584-016-1825-8 (2016).

149 Liu, L. *et al.* Effects of experimental warming and nitrogen addition on soil respiration and CH_4 fluxes from crop rotations of winter wheat–soybean/fallow. *Agricultural and Forest Meteorology* **207**, 38-47, doi:10.1016/j.agrformet.2015.03.013 (2015).

150 Liu, Q. *et al.* Belowground responses of *Picea asperata* seedlings to warming and nitrogen fertilization in the eastern Tibetan Plateau. *Ecological Research* **26**, 637-648, doi:10.1007/s11284-011-0824-5 (2011).

151 Liu, T., Xu, Z.-Z., Hou, Y.-H. & Zhou, G.-S. Effects of warming and changing precipitation rates on soil

respiration over two years in a desert steppe of northern China. *Plant and Soil* **400**, 15-27, doi:10.1007/s11104-015-2705-0 (2015).

152 Liu, W., Zhang, Z. H. E. & Wan, S. Predominant role of water in regulating soil and microbial respiration and their responses to climate change in a semiarid grassland. *Global Change Biology* **15**, 184-195, doi:10.1111/j.1365-2486.2008.01728.x (2009).

153 Liu, X. *et al.* Will nitrogen deposition mitigate warming-increased soil respiration in a young subtropical plantation? *Agricultural and Forest Meteorology* **246**, 78-85, doi:10.1016/j.agrformet.2017.06.010 (2017).

154 Liu, Y. *et al.* Short-term responses of microbial community and functioning to experimental CO_2 enrichment and warming in a Chinese paddy field. *Soil Biology and Biochemistry* **77**, 58-68, doi:10.1016/j.soilbio.2014.06.011 (2014).

155 Liu, Y. *et al.* Differential responses of soil respiration to soil warming and experimental throughfall reduction in a transitional oak forest in central China. *Agricultural and Forest Meteorology* **226-227**, 186-198, doi:10.1016/j.agrformet.2016.06.003 (2016).

156 Liu, Y. *et al.* Effects of experimental throughfall reduction and soil warming on fine root biomass and its decomposition in a warm temperate oak forest. *Science of The Total Environment* **574**, 1448-1455, doi:10.1016/j.scitotenv.2016.08.116 (2017).

157 Liu, Y. *et al.* Responses of methanogenic and methanotrophic communities to elevated atmospheric CO_2 and temperature in a paddy field. *Front Microbiol* **7**, 1895, doi:10.3389/fmicb.2016.01895 (2016).

158 Liu, Y. *et al.* Short-term response of nitrifier communities and potential nitrification activity to elevated CO_2 and temperature interaction in a Chinese paddy field. *Applied Soil Ecology* **96**, 88-98, doi:10.1016/j.apsoil.2015.06.006 (2015).

159 Lu, S., Wang, Q., Katahata, S., Naramoto, M. & Mizunaga, H. Soil microbial activities in beech forests under natural incubation conditions as affected by global warming. *Pedosphere* **24**, 709-721, doi:10.1016/s1002-0160(14)60058-8 (2014).

160 Luan, J. *et al.* Different effects of warming and cooling on the decomposition of soil organic matter in warm–temperate oak forests: a reciprocal translocation experiment. *Biogeochemistry* **121**, 551-564, doi:10.1007/s10533-014-0022-y (2014).

161 Luo, C. *et al.* Effect of warming and grazing on litter mass loss and temperature sensitivity of litter and dung mass loss on the Tibetan plateau. *Global Change Biology* **16**, 1606-1617, doi:10.1111/j.1365-2486.2009.02026.x (2010).

162 Luo, C. *et al.* Effects of grazing and experimental warming on DOC concentrations in the soil solution on the Qinghai-Tibet plateau. *Soil Biology and Biochemistry* **41**, 2493-2500, doi:10.1016/j.soilbio.2009.09.006 (2009).

163 Luo, Y., Sherry, R., Zhou, X. & Wan, S. Terrestrial carbon-cycle feedback to climate warming: experimental evidence on plant regulation and impacts of biofuel feedstock harvest. *GCB Bioenergy* **1**, 62-74, doi:10.1111/j.1757-1707.2008.01005.x (2009).

164 Ma, S. *et al.* Warming decreased and grazing increased plant uptake of amino acids in an alpine meadow. *Ecol Evol* **5**, 3995-4005, doi:10.1002/ece3.1646 (2015).

165 Marchand, F. L. *et al.* Increased turnover but little change in the carbon balance of High-Arctic tundra exposed to whole growing season warming. *Arctic Antarctic and Alpine Research* **36**, 298-307, doi:10.1657/1523-0430(2004)036[0298:Itblci]2.0.Co;2 (2004).

166 Matsunami, T., Otera, M., Amemiya, S., Kokubun, M. & Okada, M. Effect of CO_2 concentration, temperature and N fertilization on biomass production of Soybean genotypes differing in N fixation capacity. *Plant Production Science* **12**, 156-167, doi:10.1626/pps.12.156 (2009).

167 McDaniel, M. D., Kaye, J. P., Kaye, M. W. & Bruns, M. A. Climate change interactions affect soil carbon dioxide efflux and microbial functioning in a post-harvest forest. *Oecologia* **174**, 1437-1448, doi:10.1007/s00442-013-2845-y (2014).

168 McHale, P. J., Mitchell, M. J. & Bowles, F. P. Soil warming in a northern hardwood forest: trace gas fluxes and leaf litter decomposition. *Canadian Journal of Forest Research-Revue Canadienne De Recherche Forestiere* **28**, 1365-1372, doi:10.1139/cjfr-28-9-1365 (1998).

169 Melillo, J. M. *et al.* Soil warming, carbon-nitrogen interactions, and forest carbon budgets. *Proc Natl Acad Sci U S A* **108**, 9508-9512, doi:10.1073/pnas.1018189108 (2011).

170 Melillo, J. M. *et al.* Soil warming and carbon-cycle feedbacks to the climate system. *Science* **298**, 2173-2176, doi:10.1126/science.1074153 (2002).

171 Michelsen, A., Jonasson, S., Sleep, D., Havstrom, M. & Callaghan, T. V. Shoot biomass, delta C-13, nitrogen and chlorophyll responses of two arctic dwarf shrubs to in situ shading, nutrient application and warming simulating climatic change. *Oecologia* **105**, 1-12, doi:10.1007/bf00328785 (1996).

172 Morgado, L. N. *et al.* Summer temperature increase has distinct effects on the ectomycorrhizal fungal communities of moist tussock and dry tundra in Arctic Alaska. *Global Change Biology* **21**, 959-972, doi:10.1111/gcb.12716 (2015).

173 Morgan, J. A. *et al.* C4 grasses prosper as carbon dioxide eliminates desiccation in warmed semi-arid grassland. *Nature* **476**, 202-205, doi:10.1038/nature10274 (2011).

174 Natali, S. M. *et al.* Permafrost thaw and soil moisture driving CO_2 and CH_4 release from upland tundra. *Journal of Geophysical Research: Biogeosciences* **120**, 525-537, doi:10.1002/2014jg002872 (2015).

175 Nie, M. *et al.* Positive climate feedbacks of soil microbial communities in a semi-arid grassland. *Ecol Lett* **16**, 234-241, doi:10.1111/ele.12034 (2013).

176 Nielsen, C. S. *et al.* Correlations between substrate availability, dissolved CH_4, and CH_4 emissions in an arctic wetland subject to warming and plant removal. *Journal of Geophysical Research: Biogeosciences* **122**, 645-660, doi:10.1002/2016jg003511 (2017).

177 Niinistö, S. M., Silvola, J. & Kellomäki, S. Soil CO_2 efflux in a boreal pine forest under atmospheric CO_2 enrichment and air warming. *Global Change Biology* **10**, 1363-1376, doi:10.1111/j.1365-2486.2004.00799.x (2004).

178 Niu, S., Sherry, R. A., Zhou, X. & Luo, Y. Ecosystem carbon fluxes in response to warming and clipping in a tallgrass prairie. *Ecosystems* **16**, 948-961, doi:10.1007/s10021-013-9661-4 (2013).

179 Niu, S. *et al.* Water-mediated responses of ecosystem carbon fluxes to climatic change in a temperate steppe. *New Phytol* **177**, 209-219, doi:10.1111/j.1469-8137.2007.02237.x (2008).

180 Noh, N. J. *et al.* Effects of experimental warming on soil respiration and biomass in *Quercus variabilis* Blume and *Pinus densiflora* Sieb. *et* Zucc. seedlings. *Annals of Forest Science* **73**, 533-545, doi:10.1007/s13595-016-0547-4 (2016).

181 Norby, R. J., Long, T. M., Hartz-Rubin, J. S. & O'Neill, E. G. Nitrogen resorption in senescing tree leaves in a warmer, CO_2-enriched atmosephere. *Plant and Soil* **224**, 15-29, doi:10.1023/a:1004629231766 (2000).

182 Norby, R. J. & Luo, Y. Evaluating ecosystem responses to rising atmospheric CO_2 and global warming in a multi-factor world. *New Phytologist* **162**, 281-293, doi:10.1111/j.1469-8137.2004.01047.x (2004).

183 Oberbauer, S. F. *et al.* Tundra CO_2 fluxes in response to experimental warming across latitudinal and moisture gradients. *Ecological Monographs* **77**, 221-238, doi:10.1890/06-0649 (2007).

184 Olszyk, D. M. *et al.* Whole-seedling biomass allocation, leaf area, and tissue chemistry for Douglas-fir exposed to elevated CO_2 and temperature for 4 years. *Canadian Journal of Forest Research* **33**, 269-278, doi:10.1139/x02-186 (2003).

185 Osanai, Y., Janes, J. K., Newton, P. C. D. & Hovenden, M. J. Warming and elevated CO_2 combine to increase microbial mineralisation of soil organic matter. *Soil Biology and Biochemistry* **85**, 110-118, doi:10.1016/j.soilbio.2015.02.032 (2015).

186 Pajari, B. Soil respiration in a poor upland site of Scots pine stand subjected to elevated temperatures and atmospheric carbon concentration. *Plant and Soil* **168-169**, 563-570, doi:10.1007/bf00029369 (1995).

187 Pearson, M. *et al.* Effects of temperature rise and water-table-level drawdown on greenhouse gas fluxes of boreal sedge fens. *Boreal Environment Research* **20**, 489-505 (2015).

188 Peltoniemi, K. *et al.* Responses of methanogenic and methanotrophic communities to warming in varying moisture regimes of two boreal fens. *Soil Biology and Biochemistry* **97**, 144-156, doi:10.1016/j.soilbio.2016.03.007 (2016).

189 Peltoniemi, K. *et al.* Microbial ecology in a future climate: effects of temperature and moisture on microbial communities of two boreal fens. *FEMS Microbiol Ecol* **91**, doi:10.1093/femsec/fiv062 (2015).

190 Pendall, E., Osanai, Y. U. I., Williams, A. L. & Hovenden, M. J. Soil carbon storage under simulated climate change is mediated by plant functional type. *Global Change Biology* **17**, 505-514, doi:10.1111/j.1365-2486.2010.02296.x (2011).

191 Peng, F. *et al.* Intensified plant N and C pool with more available nitrogen under experimental warming in an alpine meadow ecosystem. *Ecol Evol* **6**, 8546-8555, doi:10.1002/ece3.2583 (2016).

192 Peng, F., Xue, X., You, Q., Zhou, X. & Wang, T. Warming effects on carbon release in a permafrost area of Qinghai-Tibet Plateau. *Environmental Earth Sciences* **73**, 57-66, doi:10.1007/s12665-014-3394-3 (2014).

193 Peng, F. *et al.* Effects of warming and clipping on ecosystem carbon fluxes across two hydrologically contrasting years in an alpine meadow of the Qinghai-Tibet Plateau. *PLoS One* **9**, e109319, doi:10.1371/journal.pone.0109319 (2014).

194 Peng, F., You, Q., Xue, X., Guo, J. & Wang, T. Evapotranspiration and its source components change under experimental warming in alpine meadow ecosystem on the Qinghai-Tibet plateau. *Ecological Engineering* **84**, 653-659, doi:10.1016/j.ecoleng.2015.09.069 (2015).

195 PeÑUelas, J. *et al.* Response of plant species richness and primary productivity in shrublands along a north–south gradient in Europe to seven years of experimental warming and drought: reductions in primary productivity in the heat and drought year of 2003. *Global Change Biology* **13**, 2563-2581, doi:10.1111/j.1365-2486.2007.01464.x (2007).

196 Peterjohn, W. T., Melillo, J. M., Bowles, F. P. & Steudler, P. A. Soil warming and trace gas fluxes: experimental design and preliminary flux results. *Oecologia* **93**, 18-24, doi:10.1007/bf00321185 (1993).

197 Piggott, J. J., Salis, R. K., Lear, G., Townsend, C. R. & Matthaei, C. D. Climate warming and agricultural stressors interact to determine stream periphyton community composition. *Glob Chang Biol* **21**, 206-222, doi:10.1111/gcb.12661 (2015).

198 Press, M. C., Potter, J. A., Burke, M. J. W., Callaghan, T. V. & Lee, J. A. Responses of a subarctic dwarf shrub heath community to simulated environmental change. *Journal of Ecology* **86**, 315-327, doi:10.1046/j.1365-2745.1998.00261.x (1998).

199 Pugliese, M., Gullino, M. L. & Garibaldi, A. Effects of elevated CO_2 and temperature on interactions of grapevine and powdery mildew: first results under phytotron conditions. *Journal of Plant Diseases and Protection* **117**, 9-14 (2010).

200 Pugliese, M., Liu, J., Titone, P., Garibaldi, A. & Gullino, M. L. Effects of elevated CO_2 and temperature on interactions of zucchini and powdery mildew. *Phytopathologia Mediterranea* **51**, 480-487 (2012).

201 Qiao, M. *et al.* Experimental warming effects on root nitrogen absorption and mycorrhizal infection in a subalpine coniferous forest. *Scandinavian Journal of Forest Research* **31**, 347-354,

doi:10.1080/02827581.2015.1080295 (2015).

202 Qin, Y., Yi, S., Chen, J., Ren, S. & Wang, X. Responses of ecosystem respiration to short-term experimental warming in the alpine meadow ecosystem of a permafrost site on the Qinghai–Tibetan Plateau. *Cold Regions Science and Technology* **115**, 77-84, doi:10.1016/j.coldregions.2015.03.012 (2015).

203 Quentin, A. G., Crous, K. Y., Barton, C. V. & Ellsworth, D. S. Photosynthetic enhancement by elevated CO_2 depends on seasonal temperatures for warmed and non-warmed Eucalyptus globulus trees. *Tree Physiol* **35**, 1249-1263, doi:10.1093/treephys/tpv110 (2015).

204 Rakshit, R., Patra, A. K., Pal, D., Kumar, M. & Singh, R. Effect of elevated CO_2 and temperature on nitrogen dynamics and microbial activity during wheat (*Triticum aestivum* L.) growth on a subtropical inceptisol in India. *Journal of Agronomy and Crop Science* **198**, 452-465, doi:10.1111/j.1439-037X.2012.00516.x (2012).

205 Rasheed, M. U. *et al.* The responses of shoot-root-rhizosphere continuum to simultaneous fertilizer addition, warming, ozone and herbivory in young Scots pine seedlings in a high latitude field experiment. *Soil Biology and Biochemistry* **114**, 279-294, doi:10.1016/j.soilbio.2017.07.024 (2017).

206 Ravn, N. R., Ambus, P. & Michelsen, A. Impact of decade-long warming, nutrient addition and shading on emission and carbon isotopic composition of CO_2 from two subarctic dwarf shrub heaths. *Soil Biology and Biochemistry* **111**, 15-24, doi:10.1016/j.soilbio.2017.03.016 (2017).

207 Reich, P. B. *et al.* Geographic range predicts photosynthetic and growth response to warming in co-occurring tree species. *Nature Climate Change* **5**, 148-152, doi:10.1038/nclimate2497 (2015).

208 Reynaud, S. *et al.* Interacting effects of CO_2 partial pressure and temperature on photosynthesis and calcification in a scleractinian coral. *Global Change Biology* **9**, 1660-1668, doi:10.1046/j.1365-2486.2003.00678.x (2003).

209 Richardson, S. J., Press, M. C., Parsons, A. N. & Hartley, S. E. How do nutrients and warming impact on plant communities and their insect herbivores? A 9-year study from a sub-Arctic heath. *Journal of Ecology* **90**, 544-556, doi:10.1046/j.1365-2745.2002.00681.x (2002).

210 Rillig, M. C., Wright, S. F., Shaw, M. R. & Field, C. B. Artificial climate warming positively affects arbuscular mycorrhizae but decreases soil aggregate water stability in an annual grassland. *Oikos* **97**, 52-58, doi:10.1034/j.1600-0706.2002.970105.x (2002).

211 Rinnan, R., Michelsen, A., Bååth, E. & Jonasson, S. Mineralization and carbon turnover in subarctic heath soil as affected by warming and additional litter. *Soil Biology and Biochemistry* **39**, 3014-3023, doi:10.1016/j.soilbio.2007.05.035 (2007).

212 Rinnan, R., Michelsen, A., BÅÅTh, E. & Jonasson, S. Fifteen years of climate change manipulations alter soil microbial communities in a subarctic heath ecosystem. *Global Change Biology* **13**, 28-39, doi:10.1111/j.1365-2486.2006.01263.x (2007).

213 Rinnan, R., Michelsen, A. & Jonasson, S. Effects of litter addition and warming on soil carbon, nutrient pools and microbial communities in a subarctic heath ecosystem. *Applied Soil Ecology* **39**, 271-281, doi:10.1016/j.apsoil.2007.12.014 (2008).

214 Rinnan, R., Stark, S. & Tolvanen, A. Responses of vegetation and soil microbial communities to warming and simulated herbivory in a subarctic heath. *Journal of Ecology* **97**, 788-800, doi:10.1111/j.1365-2745.2009.01506.x (2009).

215 Ruess, L., Michelsen, A., Schmidt, I. K. & Jonasson, S. Simulated climate change affecting microorganisms, nematode density and biodiversity in subarctic soils. *Plant and Soil* **212**, 63-73, doi:10.1023/a:1004567816355 (1999).

216 Ruiz-Vera, U. M., Siebers, M. H., Drag, D. W., Ort, D. R. & Bernacchi, C. J. Canopy warming caused photosynthetic acclimation and reduced seed yield in maize grown at ambient and elevated [CO_2]. *Glob*

Chang Biol **21**, 4237-4249, doi:10.1111/gcb.13013 (2015).
217 Rustad, L. E. & Fernandez, I. J. Experimental soil warming effects on CO_2 and CH_4 flux from a low elevation spruce-fir forest soil in Maine, USA. *Global Change Biology* **4**, 597-605, doi:10.1046/j.1365-2486.1998.00169.x (1998).
218 Ryan, E. M. *et al.* Gross primary production responses to warming, elevated CO_2, and irrigation: quantifying the drivers of ecosystem physiology in a semiarid grassland. *Glob Chang Biol* **23**, 3092-3106, doi:10.1111/gcb.13602 (2017).
219 Ryan, E. M. *et al.* Antecedent moisture and temperature conditions modulate the response of ecosystem respiration to elevated CO_2 and warming. *Glob Chang Biol* **21**, 2588-2602, doi:10.1111/gcb.12910 (2015).
220 Sager, E. P. S. & Hutchinson, T. C. The effects of UV-B, nitrogen fertilization, and springtime warming on sugar maple seedlings and the soil chemistry of two central Ontario forests. *Canadian Journal of Forest Research* **35**, 2432-2446, doi:10.1139/x05-160 (2005).
221 Saleska, S. R. *et al.* Plant community composition mediates both large transient decline and predicted long-term recovery of soil carbon under climate warming. *Global Biogeochemical Cycles* **16**, 3-1-3-18, doi:10.1029/2001gb001573 (2002).
222 Sardans, J., Penuelas, J. & Estiarte, M. Warming and drought change trace element bioaccumulation patterns in a Mediterranean shrubland. *Chemosphere* **70**, 874-885, doi:10.1016/j.chemosphere.2007.06.085 (2008).
223 Sardans, J., Peñuelas, J. & Estiarte, M. Warming and drought alter soil phosphatase activity and soil P availability in a Mediterranean shrubland. *Plant and Soil* **289**, 227-238, doi:10.1007/s11104-006-9131-2 (2006).
224 Sardans, J., Peñuelas, J. & Estiarte, M. Changes in soil enzymes related to C and N cycle and in soil C and N content under prolonged warming and drought in a Mediterranean shrubland. *Applied Soil Ecology* **39**, 223-235, doi:10.1016/j.apsoil.2007.12.011 (2008).
225 Schindlbacher, A. *et al.* Experimental warming effects on the microbial community of a temperate mountain forest soil. *Soil Biol Biochem* **43**, 1417-1425, doi:10.1016/j.soilbio.2011.03.005 (2011).
226 Schindlbacher, A., Zechmeister-Boltenstern, S. & Jandl, R. Carbon losses due to soil warming: Do autotrophic and heterotrophic soil respiration respond equally? *Global Change Biology* **15**, 901-913, doi:10.1111/j.1365-2486.2008.01757.x (2009).
227 Schnecker, J., Borken, W., Schindlbacher, A. & Wanek, W. Little effects on soil organic matter chemistry of density fractions after seven years of forest soil warming. *Soil Biol Biochem* **103**, 300-307, doi:10.1016/j.soilbio.2016.09.003 (2016).
228 Seo, J., Jang, I., Jung, J. Y., Lee, Y. K. & Kang, H. Warming and increased precipitation enhance phenol oxidase activity in soil while warming induces drought stress in vegetation of an Arctic ecosystem. *Geoderma* **259-260**, 347-353, doi:10.1016/j.geoderma.2015.03.017 (2015).
229 Sershen, Mdamba, B. & Ramdhani, S. Propagule and seedling responses of three species naturalised in subtropical South Africa to elevated temperatures. *Flora* **229**, 80-91, doi:10.1016/j.flora.2017.02.013 (2017).
230 Shah, N. H. & Paulsen, G. M. Interaction of drought and high temperature on photosynthesis and grain-filling of wheat. *Plant and Soil* **257**, 219-226, doi:10.1023/a:1026237816578 (2003).
231 Sharkhuu, A. *et al.* Effects of open-top passive warming chambers on soil respiration in the semi-arid steppe to taiga forest transition zone in Northern Mongolia. *Biogeochemistry* **115**, 333-348, doi:10.1007/s10533-013-9839-z (2013).
232 Sharwood, R. E., Crous, K. Y., Whitney, S. M., Ellsworth, D. S. & Ghannoum, O. Linking photosynthesis and leaf N allocation under future elevated CO_2 and climate warming in *Eucalyptus globulus*. *J Exp Bot* **68**, 1157-1167, doi:10.1093/jxb/erw484 (2017).

233 Shaw, M. R. & Harte, J. Control of litter decomposition in a subalpine meadow-sagebrush steppe ecotone under climate change. *Ecological Applications* **11**, 1206-1223, doi:10.2307/3061022 (2001).
234 Shaw, M. R. & Harte, J. Response of nitrogen cycling to simulated climate change: differential responses along a subalpine ecotone. *Global Change Biology* **7**, 193-210, doi:10.1046/j.1365-2486.2001.00390.x (2001).
235 Shaw, M. R. *et al.* Grassland responses to global environmental changes suppressed by elevated CO_2. *Science* **298**, 1987-1990, doi:10.1126/science.1075312 (2002).
236 Shen, W., Reynolds, J. F. & Hui, D. Responses of dryland soil respiration and soil carbon pool size to abrupt vs. gradual and individual vs. combined changes in soil temperature, precipitation, and atmospheric [CO_2]: a simulation analysis. *Global Change Biology* **15**, 2274-2294, doi:10.1111/j.1365-2486.2009.01857.x (2009).
237 Shi, F., Chen, H., Chen, H., Wu, Y. & Wu, N. The combined effects of warming and drying suppress CO_2 and N_2O emission rates in an alpine meadow of the eastern Tibetan Plateau. *Ecological Research* **27**, 725-733, doi:10.1007/s11284-012-0950-8 (2012).
238 Shi, F., Wu, N., Wu, Y. & Wang, Q. Effect of simulated temperature enhancement on growth and photosynthesis of *Deschampsia caespitosa* and *Thlaspi arvense* in Northwestern Sichuan, China. *Chinese Journal of Appplied Environmental Biology* **2009**, 750-755, doi:10.3724/sp.J.1145.2009.00750 (2010).
239 Shi, F.-S., Che, H.-F. & Wu, N. Effect of Experimental warming on carbon and nitrogen Content of Sub-alpine meadow in Northwestern Sichuan. *Bulletin of Botanical Research* **28**, 730-736 (2008).
240 Shi, F.-S., Wu, N. & Wu, Y. Responses of plant growth and substance allocation of three dominant plant species to experimental warming in an alpine grassland, Northwestern Sichuan, China. *Chinese Journal of Plant Ecology* **34**, 488-497, doi:10.3773/j.issn.1005-264x.2010.05.002 (2010).
241 Sistla, S. A. *et al.* Long-term warming restructures Arctic tundra without changing net soil carbon storage. *Nature* **497**, 615-618, doi:10.1038/nature12129 (2013).
242 Sjogersten, S., van der Wal, R. & Woodin, S. J. Habitat type determines herbivory controls over CO_2 fluxes in a warmer arctic. *Ecology* **89**, 2103-2116, doi:10.1890/07-1601.1 (2008).
243 Song, B., Niu, S. & Wan, S. Precipitation regulates plant gas exchange and its long-term response to climate change in a temperate grassland. *Journal of Plant Ecology* **9**, 531-541, doi:10.1093/jpe/rtw010 (2016).
244 Song, U. Temperature-dependent performance of competitive native and alien invasive plant species. *Acta Oecologica* **84**, 8-14, doi:10.1016/j.actao.2017.08.001 (2017).
245 Sorensen, P. L., Michelsen, A. & Jonasson, S. Nitrogen uptake during one year in subarctic plant functional groups and in microbes after long-term warming and fertilization. *Ecosystems* **11**, 1223-1233, doi:10.1007/s10021-008-9204-6 (2008).
246 Sorokin, Y., Zelikova, T. J., Blumenthal, D., Williams, D. G. & Pendall, E. Seasonally contrasting responses of evapotranspiration to warming and elevated CO_2 in a semiarid grassland. *Ecohydrology* **10**, doi:10.1002/eco.1880 (2017).
247 Sowerby, A. *et al.* Microbial community changes in heathland soil communities along a geographical gradient: interaction with climate change manipulations. *Soil Biology and Biochemistry* **37**, 1805-1813, doi:10.1016/j.soilbio.2005.02.023 (2005).
248 Strebel, D., Elberling, B., Morgner, E., Knicker, H. E. & Cooper, E. J. Cold-season soil respiration in response to grazing and warming in High-Arctic Svalbard. *Polar Research* **29**, 46-57, doi:10.1111/j.1751-8369.2010.00154.x (2016).
249 Streit, K. *et al.* Soil warming alters microbial substrate use in alpine soils. *Glob Chang Biol* **20**, 1327-1338, doi:10.1111/gcb.12396 (2014).
250 Sullivan, P. F., Arens, S. J. T., Chimner, R. A. & Welker, J. M. Temperature and microtopography interact to

control carbon cycling in a high arctic fen. *Ecosystems* **11**, 61-76, doi:10.1007/s10021-007-9107-y (2007).

251 Sun, D.-d. *et al.* Effects of experimental warming on soil microbial communities in two contrasting subalpine forest ecosystems, eastern Tibetan Plateau, China. *Journal of Mountain Science* **13**, 1442-1452, doi:10.1007/s11629-015-3733-1 (2016).

252 Suseela, V., Conant, R. T., Wallenstein, M. D. & Dukes, J. S. Effects of soil moisture on the temperature sensitivity of heterotrophic respiration vary seasonally in an old-field climate change experiment. *Global Change Biology* **18**, 336-348, doi:10.1111/j.1365-2486.2011.02516.x (2012).

253 Suzuki, M. *et al.* Effects of long-term experimental warming on plants and soil microbes in a cool temperate semi-natural grassland in Japan. *Ecological Research* **31**, 957-962, doi:10.1007/s11284-016-1386-3 (2016).

254 Tang, Z., Yin, H., Zhou, X., Wei, Y. & Liu, Q. Short-term effects of night warming and nitrogen addition on soil respiration of subalpine coniferous, western Sichuan, China. *Chinese Journal of Appplied Environmental Biology* **18**, doi:10.3724/sp.J.1145.2012.00713 (2012).

255 Tiiva, P. *et al.* Climatic warming increases isoprene emission from a subarctic heath. *New Phytol* **180**, 853-863, doi:10.1111/j.1469-8137.2008.02587.x (2008).

256 Tokida, T. *et al.* The contribution of entrapped gas bubbles to the soil methane pool and their role in methane emission from rice paddy soil in free-air [CO_2] enrichment and soil warming experiments. *Plant and Soil* **364**, 131-143, doi:10.1007/s11104-012-1356-7 (2012).

257 Tscherko, D., Kandeler, E. & Jones, T. H. Effect of temperature on below-ground N-dynamics in a weedy model ecosystem at ambient and elevated atmospheric CO_2 levels. *Soil Biology & Biochemistry* **33**, 491-501, doi:10.1016/s0038-0717(00)00190-5 (2001).

258 Tu, C. & Li, F. Responses of greenhouse gas fluxes to experimental warming in wheat season under conventional tillage and no-tillage fields. *J Environ Sci (China)* **54**, 314-327, doi:10.1016/j.jes.2016.09.016 (2017).

259 Tu, C. *et al.* Effect of experimental warming on soil respiration under conventional tillage and no-tillage farmland in the North China Plain. *Journal of Integrative Agriculture* **16**, 967-979, doi:10.1016/s2095-3119(16)61449-1 (2017).

260 Updegraff, K., Bridgham, S. D., Pastor, J., Weishampel, P. & Harth, C. Response of CO_2 and CH_4 emissions from peatlands to warming and water table manipulation. *Ecological Applications* **11**, 311-326 (2001).

261 Usami, T., Lee, J. & Oikawa, T. Interactive effects of increased temperature and CO_2 on the growth of Quercus myrsinaefolia saplings. *Plant Cell and Environment* **24**, 1007-1019, doi:10.1046/j.1365-3040.2001.00753.x (2001).

262 Uselman, S. M., Qualls, R. G. & Thomas, R. B. Effects of increased atmospheric CO_2, temperature, and soil N availability on root exudation of dissolved organic carbon by a N-fixing tree (*Robinia pseudoacacia* L.). *Plant and Soil* **222**, 191-202, doi:10.1023/a:1004705416108 (2000).

263 van Meeteren, M. J. M., Tietema, A., van Loon, E. E. & Verstraten, J. M. Microbial dynamics and litter decomposition under a changed climate in a Dutch heathland. *Applied Soil Ecology* **38**, 119-127, doi:10.1016/j.apsoil.2007.09.006 (2008).

264 Veteli, T. O., Kuokkanen, K., Julkunen-Tiitto, R., Roininen, H. & Tahvanainen, J. Effects of elevated CO_2 and temperature on plant growth and herbivore defensive chemistry. *Global Change Biology* **8**, 1240-1252, doi:10.1046/j.1365-2486.2002.00553.x (2002).

265 Vicente-Serrano, S. M. *et al.* Evapotranspiration deficit controls net primary production and growth of silver fir: Implications for Circum-Mediterranean forests under forecasted warmer and drier conditions. *Agricultural and Forest Meteorology* **206**, 45-54, doi:10.1016/j.agrformet.2015.02.017 (2015).

266 Vogan, P. J. & Sage, R. F. Effects of low atmospheric CO_2 and elevated temperature during growth on the

gas exchange responses of C3, C3-C4 intermediate, and C4 species from three evolutionary lineages of C4 photosynthesis. *Oecologia* **169**, 341-352, doi:10.1007/s00442-011-2201-z (2012).

267 Vogel, J. G., Bronson, D., Gower, S. T. & Schuur, E. A. G. The response of root and microbial respiration to the experimental warming of a boreal black spruce forest. *Canadian Journal of Forest Research* **44**, 986-993, doi:10.1139/cjfr-2014-0056 (2014).

268 Voigt, C. *et al.* Warming of subarctic tundra increases emissions of all three important greenhouse gases - carbon dioxide, methane, and nitrous oxide. *Glob Chang Biol* **23**, 3121-3138, doi:10.1111/gcb.13563 (2017).

269 Volder, A. *et al.* Does greater night-time, rather than constant, warming alter growth of managed pasture under under ambient and elevated atmospheric CO2? *New Phytologist* **162**, 397-411, doi:10.1111/j.1469-8137.2004.01025.x (2004).

270 Volder, A., Gifford, R. M. & Evans, J. R. Effects of elevated atmospheric CO_2, cutting frequency, and differential day/night atmospheric warming on root growth and turnover of Phalaris swards. *Global Change Biology* **13**, 1040-1052, doi:10.1111/j.1365-2486.2007.01321.x (2007).

271 Volder, A., Gifford, R. M. & Evans, J. R. Effects of elevated atmospheric CO_2 concentrations, clipping regimen and differential day/night atmospheric warming on tissue nitrogen concentrations of a perennial pasture grass. *AoB Plants* **7**, doi:10.1093/aobpla/plv094 (2015).

272 Vu, J. C. V., Allen, L. H., Boote, K. J. & Bowes, G. Effects of elevated CO_2 and temperature on photosynthesis and Rubisco in rice and soybean. *Plant Cell and Environment* **20**, 68-76, doi:10.1046/j.1365-3040.1997.d01-10.x (1997).

273 Wan, S., Hui, D., Wallace, L. & Luo, Y. Direct and indirect effects of experimental warming on ecosystem carbon processes in a tallgrass prairie. *Global Biogeochemical Cycles* **19**, GB2014, doi:10.1029/2004gb002315 (2005).

274 Wan, S., Norby, R. J., Ledford, J. & Weltzin, J. F. Responses of soil respiration to elevated CO_2, air warming, and changing soil water availability in a model old-field grassland. *Global Change Biology* **13**, 2411-2424, doi:10.1111/j.1365-2486.2007.01433.x (2007).

275 Wan, S., Norby, R. J., Pregitzer, K. S., Ledford, J. & O'Neill, E. G. CO_2 enrichment and warming of the atmosphere enhance both productivity and mortality of maple tree fine roots. *New Phytologist* **162**, 437-446, doi:10.1111/j.1469-8137.2004.01034.x (2004).

276 Wang, C. *et al.* Effects of long-term experimental warming on plant community properties and soil microbial community composition in an alpine meadow. *Israel Journal of Ecology & Evolution*, 1-12, doi:10.1080/15659801.2017.1281201 (2017).

277 Wang, C. *et al.* The effect of simulated warming on root dynamics and soil microbial community in an alpine meadow of the Qinghai-Tibet Plateau. *Applied Soil Ecology* **116**, 30-41, doi:10.1016/j.apsoil.2017.03.005 (2017).

278 Wang, D., Heckathorn, S. A., Mainali, K. & Hamilton, E. W. Effects of N on plant response to heat-wave: a field study with prairie vegetation. *J Integr Plant Biol* **50**, 1416-1425, doi:10.1111/j.1744-7909.2008.00748.x (2008).

279 Wang, K., Kellomaki, S. & Laitinen, K. Effect of needle age, long-term temperature and CO_2 treatments on the photosynthesis of Scots pine. *Tree Physiology* **15**, 211-218, doi:10.1093/treephys/15.4.211 (1995).

280 Wang, X., Nakatsubo, T. & Nakane, K. Impacts of elevated CO_2 and temperature on soil respiration in warm temperate evergreen *Quercus glauca* stands: an open-top chamber experiment. *Ecological Research* **27**, 595-602, doi:10.1007/s11284-012-0932-x (2012).

281 Ward, S. E. *et al.* Warming effects on greenhouse gas fluxes in peatlands are modulated by vegetation composition. *Ecol Lett* **16**, 1285-1293, doi:10.1111/ele.12167 (2013).

282 Wayne, P. M., Reekie, E. G. & Bazzaz, F. A. Elevated CO_2 ameliorates birch response to high temperature and frost stress: implications for modeling climate-induced geographic range shifts. *Oecologia* **114**, 335-342, doi:10.1007/s004420050455 (1998).

283 Webb, E. E. ***et al.*** Increased wintertime CO2 loss as a result of sustained tundra warming. *Journal of Geophysical Research: Biogeosciences* **121**, 249-265, doi:10.1002/2014jg002795 (2016).

284 Welker, J. M., Fahnestock, J. T., Henry, G. H. R., O'Dea, K. W. & Chimner, R. A. CO_2 exchange in three Canadian High Arctic ecosystems: response to long-term experimental warming. *Global Change Biology* **10**, 1981-1995, doi:10.1111/j.1365-2486.2004.00857.x (2004).

285 Weltzin, J. F. ***et al.*** Response of bog and fen plant communities to warming and water-table manipulations. *Ecology* **81**, 3464-3478, doi:10.2307/177507 (2000).

286 Wertin, T. M., McGuire, M. A. & Teskey, R. O. The influence of elevated temperature, elevated atmospheric CO_2 concentration and water stress on net photosynthesis of loblolly pine (*Pinus taeda* L.) at northern, central and southern sites in its native range. *Global Change Biology* **16**, 2089-2103, doi:10.1111/j.1365-2486.2009.02053.x (2010).

287 Wertin, T. M., McGuire, M. A., van Iersel, M., Ruter, J. M. & Teskey, R. O. Effects of elevated temperature and [CO_2] on photosynthesis, leaf respiration, and biomass accumulation of *Pinus taeda* seedlings at a cool and a warm site within the species' current range. *Canadian Journal of Forest Research* **42**, 943-957, doi:10.1139/x2012-050 (2012).

288 Wheeler, J. A., Gonzalez, N. M. & Stinson, K. A. Red hot maples: Acer rubrum first-year phenology and growth responses to soil warming. *Canadian Journal of Forest Research* **47**, 159-165, doi:10.1139/cjfr-2016-0288 (2017).

289 Wu, Y. ***et al.*** Effects of warming on root diameter, distribution, and longevity in an alpine meadow. *Plant Ecology* **215**, 1057-1066, doi:10.1007/s11258-014-0364-5 (2014).

290 Xia, J., Niu, S. & Wan, S. Response of ecosystem carbon exchange to warming and nitrogen addition during two hydrologically contrasting growing seasons in a temperate steppe. *Global Change Biology* **15**, 1544-1556, doi:10.1111/j.1365-2486.2008.01807.x (2009).

291 Xiong, J. ***et al.*** Divergent responses of soil fungi functional groups to short-term warming. *Microb Ecol* **68**, 708-715, doi:10.1007/s00248-014-0385-6 (2014).

292 Xiong, Q. ***et al.*** Warming and nitrogen deposition are interactive in shaping surface soil microbial communities near the alpine timberline zone on the eastern Qinghai–Tibet Plateau, southwestern China. *Applied Soil Ecology* **101**, 72-83, doi:10.1016/j.apsoil.2016.01.011 (2016).

293 Xu, C. Y., Salih, A., Ghannoum, O. & Tissue, D. T. Leaf structural characteristics are less important than leaf chemical properties in determining the response of leaf mass per area and photosynthesis of *Eucalyptus saligna* to industrial-age changes in [CO_2] and temperature. *J Exp Bot* **63**, 5829-5841, doi:10.1093/jxb/ers231 (2012).

294 Xu, G., Jiang, H., Zhang, Y., Korpelainen, H. & Li, C. Effect of warming on extracted soil carbon pools of *Abies faxoniana* forest at two elevations. *Forest Ecology and Management* **310**, 357-365, doi:10.1016/j.foreco.2013.08.038 (2013).

295 Xu, M. ***et al.*** Effects of warming and clipping on plant and soil properties of an alpine meadow in the Qinghai-Tibetan Plateau, China. *Journal of Arid Land* **7**, 189-204, doi:10.1007/s40333-014-0010-z (2014).

296 Xu, Z., Zhao, C., Yin, H. & Liu, Q. Warming and forest management interactively affect the decomposition of subalpine forests on the eastern Tibetan Plateau: A four-year experiment. *Geoderma* **239-240**, 223-228, doi:10.1016/j.geoderma.2014.10.018 (2015).

297 Xu, Z.-f. ***et al.*** Initial soil responses to experimental warming in two contrasting forest ecosystems, Eastern

Tibetan Plateau, China: Nutrient availabilities, microbial properties and enzyme activities. *Applied Soil Ecology* **46**, 291-299, doi:10.1016/j.apsoil.2010.07.005 (2010).

298 Yin, H. *et al.* Warming effects on root morphological and physiological traits: The potential consequences on soil C dynamics as altered root exudation. *Agricultural and Forest Meteorology* **180**, 287-296, doi:10.1016/j.agrformet.2013.06.016 (2013).

299 Yin, H. J., Liu, Q. & Lai, T. Warming effects on growth and physiology in the seedlings of the two conifers *Picea asperata* and *Abies faxoniana* under two contrasting light conditions. *Ecological Research* **23**, 459-469, doi:10.1007/s11284-007-0404-x (2007).

300 Yoon, S. T., Hoogenboom, G., Flitcroft, I. & Bannayan, M. Growth and development of cotton (*Gossypium hirsutum* L.) in response to CO_2 enrichment under two different temperature regimes. *Environmental and Experimental Botany* **67**, 178-187, doi:10.1016/j.envexpbot.2009.06.015 (2009).

301 Yoshitake, S. *et al.* Soil microbial response to experimental warming in cool temperate semi-natural grassland in Japan. *Ecological Research* **30**, 235-245, doi:10.1007/s11284-014-1209-3 (2014).

302 Yu, C. Q., Shen, Z. X., Zhang, X. Z., Sun, W. & Fu, G. Response of soil C and N, dissolved organic C and N, and inorganic N to short-term experimental warming in an Alpine meadow on the Tibetan Plateau. *Scientific World Journal* **2014**, 152576, doi:10.1155/2014/152576 (2014).

303 Zavaleta, E. S., Shaw, M. R., Chiariello, N. R., Mooney, H. A. & Field, C. B. Additive effects of simulated climate changes, elevated CO_2, and nitrogen deposition on grassland diversity. *Proceedings of the National Academy of Sciences of the United States of America* **100**, 7650-7654, doi:10.1073/pnas.0932734100 (2003).

304 Zavaleta, E. S. *et al.* Grassland responses to three years of elevated temperature, CO_2, precipitation, and N deposition. *Ecological Monographs* **73**, 585-604, doi:10.1890/02-4053 (2003).

305 Zeppel, M. J. *et al.* Nocturnal stomatal conductance responses to rising [CO_2], temperature and drought. *New Phytol* **193**, 929-938, doi:10.1111/j.1469-8137.2011.03993.x (2012).

306 Zhang, L., Zhu, L., Yu, M. & Zhong, M. Warming decreases photosynthates and yield of soybean [*Glycine max* (L.) Merrill] in the North China Plain. *The Crop Journal* **4**, 139-146, doi:10.1016/j.cj.2015.12.003 (2016).

307 Zhang, N. *et al.* Soil microbial responses to warming and increased precipitation and their implications for ecosystem C cycling. *Oecologia* **173**, 1125-1142, doi:10.1007/s00442-013-2685-9 (2013).

308 Zhang, T., Guo, R., Gao, S., Guo, J. & Sun, W. Responses of plant community composition and biomass production to warming and nitrogen deposition in a temperate meadow ecosystem. *PLoS One* **10**, e0123160, doi:10.1371/journal.pone.0123160 (2015).

309 Zhang, W. *et al.* Soil microbial responses to experimental warming and clipping in a tallgrass prairie. *Global Change Biology* **11**, 266-277, doi:10.1111/j.1365-2486.2005.00902.x (2005).

310 Zhang, X., Johnston, E. R., Li, L., Konstantinidis, K. T. & Han, X. Experimental warming reveals positive feedbacks to climate change in the Eurasian Steppe. *ISME J* **11**, 885-895, doi:10.1038/ismej.2016.180 (2017).

311 Zhang, Y. *et al.* Effects of grazing and climate warming on plant diversity, productivity and living state in the alpine rangelands and cultivated grasslands of the Qinghai-Tibetan Plateau. *The Rangeland Journal* **37**, doi:10.1071/rj14080 (2015).

312 Zhang, Z., Qiao, M., Li, D., Yin, H. & Liu, Q. Do warming-induced changes in quantity and stoichiometry of root exudation promote soil N transformations via stimulation of soil nitrifiers, denitrifiers and ammonifiers? *European Journal of Soil Biology* **74**, 60-68, doi:10.1016/j.ejsobi.2016.03.007 (2016).

313 Zhao, Y. H. *et al.* Growth under elevated air temperature alters secondary metabolites in Robinia pseudoacacia L. seedlings in Cd- and Pb-contaminated soils. *Sci Total Environ* **565**, 586-594, doi:10.1016/j.scitotenv.2016.05.058 (2016).

314 Zhao, Z. *et al.* Effects of warming and nitrogen deposition on CH_4, CO_2 and N_2O emissions in alpine grassland ecosystems of the Qinghai-Tibetan Plateau. *Sci Total Environ* **592**, 565-572, doi:10.1016/j.scitotenv.2017.03.082 (2017).

315 Zhong, Q., Du, Q., Gong, J., Zhang, C. & Wang, K. Effects of in situ experimental air warming on the soil respiration in a coastal salt marsh reclaimed for agriculture. *Plant and Soil* **371**, 487-502, doi:10.1007/s11104-013-1707-z (2013).

316 Zhong, Z. M., Shen, Z. X. & Fu, G. Response of soil respiration to experimental warming in a highland barley of the Tibet. *Springerplus* **5**, 137, doi:10.1186/s40064-016-1761-0 (2016).

317 Zhou, X. *et al.* Soil extractable carbon and nitrogen, microbial biomass and microbial metabolic activity in response to warming and increased precipitation in a semiarid Inner Mongolian grassland. *Geoderma* **206**, 24-31, doi:10.1016/j.geoderma.2013.04.020 (2013).

318 Zhou, X. *et al.* Effects of elevated CO_2 and temperature on leaf characteristics, photosynthesis and carbon storage in aboveground biomass of a boreal bioenergy crop (*Phalaris arundinacea* L.) under varying water regimes. *GCB Bioenergy* **3**, 223-234, doi:10.1111/j.1757-1707.2010.01075.x (2011).

319 Zhou, X., Sherry, R. A., An, Y., Wallace, L. L. & Luo, Y. Main and interactive effects of warming, clipping, and doubled precipitation on soil CO_2 efflux in a grassland ecosystem. *Global Biogeochemical Cycles* **20**, n/a-n/a, doi:10.1029/2005gb002526 (2006).

320 Zhou, X., Wan, S. & Luo, Y. Source components and interannual variability of soil CO_2 efflux under experimental warming and clipping in a grassland ecosystem. *Global Change Biology* **13**, 761-775, doi:10.1111/j.1365-2486.2007.01333.x (2007).

321 Zhu, X. *et al.* Effects of warming, grazing/cutting and nitrogen fertilization on greenhouse gas fluxes during growing seasons in an alpine meadow on the Tibetan Plateau. *Agricultural and Forest Meteorology* **214-215**, 506-514, doi:10.1016/j.agrformet.2015.09.008 (2015).

322 Zong, S. *et al.* Nitrogen deposition but not climate warming promotes *Deyeuxia angustifolia* encroachment in alpine tundra of the Changbai Mountains, Northeast China. *Sci Total Environ* **544**, 85-93, doi:10.1016/j.scitotenv.2015.11.144 (2016).
