# Supplementary material for: Global systematic review with meta-analysis shows that warming effects on terrestrial plant biomass allocation are influenced by precipitation and mycorrhizal association
Source: Nat Commun. 2022 Aug 20;13:4914. doi: 10.1038/s41467-022-32671-9 (PMC9392739; doi:10.1038/s41467-022-32671-9)
Supplement: Supplementary file 2 — Description of Additional Supplementary Files [file 41467_2022_32671_MOESM2_ESM.pdf]

Title: Supplementary Data 1

Description: Database 1: Mean, standard deviation, and replicates of R/S, total biomass (TB), above- and belowground biomass (AGB and BGB) in control (C.) and warming (W.) treatment we used in this meta-analysis.

Title: Supplementary Data 2

Description: Database 2: The data we used in the analysis of structural equation model (SEM).

Title: Supplementary Data 3

Description: Database 3: The database of microbial biomass (MB C/N), microbial biomass C/N (MB C/N), soil inorganic nitrogen (SIN), Soil NH4+, Soil NO3-, and water use efficiency (WUE) we used in this study.
