# Supplementary material for: Global systematic review with meta-analysis shows that warming effects on terrestrial plant biomass allocation are influenced by precipitation and mycorrhizal association
Source: Nat Commun. 2022 Aug 20;13:4914. doi: 10.1038/s41467-022-32671-9 (PMC9392739; doi:10.1038/s41467-022-32671-9)
Supplement: Supplementary file 3 — Reporting Summary [file 41467_2022_32671_MOESM3_ESM.pdf]

nature portfolio

Corresponding author(s): Xuhui Zhou

Last updated by author(s): Aug 10, 2022

Reporting Summary

Nature Portfolio wishes to improve the reproducibility of the work that we publish. This form provides structure for consistency and transparency in reporting. For further information on Nature Portfolio policies, see our Editorial Policies and the Editorial Policy Checklist.

Statistics

For all statistical analyses, confirm that the following items are present in the figure legend, table legend, main text, or Methods section.

n/a	Confirmed	
☐	☒	The exact sample size (*n*) for each experimental group/condition, given as a discrete number and unit of measurement
☐	☒	A statement on whether measurements were taken from distinct samples or whether the same sample was measured repeatedly
☐	☒	The statistical test(s) used AND whether they are one- or two-sided<br>*Only common tests should be described solely by name; describe more complex techniques in the Methods section.*
☒	☐	A description of all covariates tested
☐	☒	A description of any assumptions or corrections, such as tests of normality and adjustment for multiple comparisons
☐	☒	A full description of the statistical parameters including central tendency (e.g. means) or other basic estimates (e.g. regression coefficient) AND variation (e.g. standard deviation) or associated estimates of uncertainty (e.g. confidence intervals)
☐	☒	For null hypothesis testing, the test statistic (e.g. *F*, *t*, *r*) with confidence intervals, effect sizes, degrees of freedom and *P* value noted<br>*Give P values as exact values whenever suitable.*
☒	☐	For Bayesian analysis, information on the choice of priors and Markov chain Monte Carlo settings
☐	☒	For hierarchical and complex designs, identification of the appropriate level for tests and full reporting of outcomes
☐	☒	Estimates of effect sizes (e.g. Cohen's *d*, Pearson's *r*), indicating how they were calculated

Our web collection on statistics for biologists contains articles on many of the points above.

Software and code

Policy information about availability of computer code

Data collection	Peer-reviewed journal articles related to plant biomass under experimental warming were searched from Web of Science (Wos, 1950-2020, Thomson Reuters, New York, NY, USA) to compile the data for meta-analysis. Databases used for search in WoS included 1) WoS Core Collection, 2) Inspec®, 3) KCI-Korean Journal Database, 4) BIOSIS Previews, 5) Derwent Innovations Index, 6) Russian Science Citation Index, 7) Data Citation Index, 8) Chinese Science Citation Database, 9) MEDLINE®, and 10) SciELO Citation Index. All the data were were extracted using software GetData (version 2.22).
Data analysis	All meta-analyses, including meta-regressions, were performed using the package 'metafor' (version 3.4-0) in R (version 4.1.3, R Core Team, 2018). The importance of each predictors for the response ratio of concerned variables was tested using the "glmulti" package (version 1.0.8) in R . Nested analysis was performed using the "nlme" package (version 3.1-158) in R. The phylogenetic signals on warming response of concerned variables was tested using the "picante" package (version 1.8.2) in R. The between-group heterogeneity (Qb) of variables' response was estimated using the Q-statistic in MetaWin 2.1. All other details of data analyses are provided in the Methods section of the main manuscript.

For manuscripts utilizing custom algorithms or software that are central to the research but not yet described in published literature, software must be made available to editors and reviewers. We strongly encourage code deposition in a community repository (e.g. GitHub). See the Nature Portfolio guidelines for submitting code & software for further information.

Data

Policy information about availability of data

All manuscripts must include a data availability statement. This statement should provide the following information, where applicable:

- Accession codes, unique identifiers, or web links for publicly available datasets
- A description of any restrictions on data availability
- For clinical datasets or third party data, please ensure that the statement adheres to our policy

All data generated or analyzed during this study are included in this published article. There were three supplementary database listed as Supplementary Data 1-3 to show: 1) The mean, standard deviation, and replicates of root: shoot ratio, total biomass, above- and belowground biomass in control and warming treatment we used in this meta-analysis; 2) The data we used in the analysis of structural equation model (SEM); 3) The database of microbial biomass, microbial biomass C/N, soil inorganic nitrogen, Soil NH4+, Soil NO3-, and water use efficiency we used in this study. In addition, the latest FungalRoot database (https://nt.ars-grin.gov/fungaldatabases/) was used to confirm the mycorrhizal fungi types of dominant plants in biomes. Global Gridded Surfaces of Selected Soil Characteristics (soil bulk density from IGBP-DIS) and Harmonized World Soil Database (CLAY and SOC, from version 1.2, https://daac.ornl.gov/SOILS/guides/HWSD.html) were used to extract climate and soil properties of study sites.

Human research participants

Policy information about studies involving human research participants and Sex and Gender in Research.

Reporting on sex and gender	not applicable
Population characteristics	not applicable
Recruitment	not applicable
Ethics oversight	not applicable

Note that full information on the approval of the study protocol must also be provided in the manuscript.

Field-specific reporting

Please select the one below that is the best fit for your research. If you are not sure, read the appropriate sections before making your selection.

☐ Life sciences ☐ Behavioural & social sciences ☒ Ecological, evolutionary & environmental sciences

For a reference copy of the document with all sections, see nature.com/documents/nr-reporting-summary-flat.pdf

Ecological, evolutionary & environmental sciences study design

All studies must disclose on these points even when the disclosure is negative.

Study description	Biomass allocation in plants is fundamental for understanding and predicting terrestrial carbon storage. Here, the authors conduct a meta-analysis showing that warming effect on plant root: shoot ratio is influenced by precipitation and the type of mycorrhizal fungi associated.
Research sample	Based on the selection criteria of studies we used, 322 papers (searched from Web of Science (Wos, 1950-2020, Thomson Reuters, New York, NY, USA)) were selected to extract the variables including plant total biomass (TB), aboveground biomass (AGB), belowground biomass (or root biomass, BGB), root: shoot ratio (R/S), and some related variables, e.g., soil moisture, water use efficiency (WUE), soil inorganic nitrogen (SIN), soil NH4+, soil NO3-, microbial biomass (MB), and/or microbial biomass C/N (MB C/N) in control and warming groups. The following criteria were conducted to select the studies compiled in database : (i) Warming treatments conducted in terrestrial biomes and at least one of above-mentioned variables were recorded. (ii) The experimental temperature, warming method (e.g., open top chamber, infrared heater, or soil heating cable and greenhouse) and dominant plant species were indicated clearly in both warming and control groups. (iii) Apart from the difference in experimental temperature, other initial environmental condition and plant species compositions were the same in the control and warming. (iv) Warming duration was at least longer than one growing season. (v) The mean, standard deviations/errors, and sample sizes of these variables could be extracted from the figures, tables or context directly. In total, 148, 272, 132, and 94 observations for TB, AGB, BGB and R/S were used to perform meta-analysis calculations, representing the central trend of warming effect on plant biomass allocation in terrestrial ecosystem.
Sampling strategy	Using the ISI Web of Science, peer-reviewed journal articles (1950-2020) related to plant biomass under experimental warming were searched with specific keywords： 1) 'warming' or 'temperature' or 'heat*' or 'greenhouse'; and, 2) 'biomass' or 'plant' or 'allocation' or 'root' or 'leaf' or 'stem' or 'photosynthe*' or 'growth' or 'aboveground' or 'belowground' or 'respiration' or 'nitrogen' or 'soil' or 'carbon' or 'microb*'. In total, 86215 records were found at the very beginning, according to PRISMA flow diagram, 322 studies were remained in this meta-analysis (Supplementary Information). Details about the inclusion/ exclusion criteria for publications were reported in data collection section of the Methods in the main manuscript. In order to confirm the sufficiency of

	data for this analysis, we used funnel plot method to test the sufficiency of data in each subgroup, e. g., in subgroup of plants with a certain type of mycorrhizal fungi association. If the mean effect had significant difference from zero, Rosenthal's fail-safe number was calculated. Since the fail-safe number was over 5n+10 (n was the actual sample size), we made conclusion that the data in this analysis of this group was sufficient.
Data collection	Searching with above-mentioned specific keywords, LYZ recorded 86215 studies first. Then the duplicate records and records that was not article, or not science technology, or not research field related to ecology, plant science and life science, or not retrieved were removed. Then, according to specific content in title, abstract, methods and variables (see the criteria in study selection), CYL and CHL excluded 20085 studies. Finally, 322 studies were included in this meta-analysis (see PRISMA flow diagram in Supplementary Information file, and Database in Supplementary Data 1-3).
Timing and spatial scale	Together, the studies published between 1993 to 2020, mainly distributed in East Asia, North America and Europe . The mean annual precipitation and temperature (MAP and MAT) of sites, warming duration and magnitude ranged from 27 mm to 2400 mm, -20 oC to 30 oC, one growing season to 25 years, and 0.26 oC to 12 oC, respectively.
Data exclusions	We excluded data from laboratory incubation studies. Other exclusion information can be obtained from the PRISMA flow diagram provided in Supplementary information file.
Reproducibility	Data collection and statistical procedures to analyze the data are detailed in the Methods section so that all our results can be reproduced. Moreover, we have provided all the code to produce the main results and figures in Figshare.
Randomization	We used random effects meta-analysis, which incorporates each study as independent and random.
Blinding	Not applicable. There were no human or animal research participants involved in this study.

Did the study involve field work? ☐ Yes ☒ No

Reporting for specific materials, systems and methods

We require information from authors about some types of materials, experimental systems and methods used in many studies. Here, indicate whether each material, system or method listed is relevant to your study. If you are not sure if a list item applies to your research, read the appropriate section before selecting a response.

Materials & experimental systems

n/a	Involved in the study	
☒	☐	Antibodies
☒	☐	Eukaryotic cell lines
☒	☐	Palaeontology and archaeology
☒	☐	Animals and other organisms
☒	☐	Clinical data
☒	☐	Dual use research of concern

Methods

n/a	Involved in the study	
☒	☐	ChIP-seq
☒	☐	Flow cytometry
☒	☐	MRI-based neuroimaging
